# Targeting genomic SARS-CoV-2 RNA with siRNAs allows efficient inhibition of viral replication and spread

**DOI:** 10.1093/nar/gkab1248

**Published:** 2021-12-20

**Authors:** Shubhankar Ambike, Cho-Chin Cheng, Martin Feuerherd, Stoyan Velkov, Domizia Baldassi, Suliman Qadir Afridi, Diana Porras-Gonzalez, Xin Wei, Philipp Hagen, Nikolaus Kneidinger, Mircea Gabriel Stoleriu, Vincent Grass, Gerald Burgstaller, Andreas Pichlmair, Olivia M Merkel, Chunkyu Ko, Thomas Michler

**Affiliations:** Institute of Virology, School of Medicine, Technische Universität München / Helmholtz Zentrum München, Trogerstr. 30, 81675 Munich, Germany; Institute of Virology, School of Medicine, Technische Universität München / Helmholtz Zentrum München, Trogerstr. 30, 81675 Munich, Germany; Institute of Virology, School of Medicine, Technische Universität München / Helmholtz Zentrum München, Trogerstr. 30, 81675 Munich, Germany; Institute of Virology, School of Medicine, Technische Universität München / Helmholtz Zentrum München, Trogerstr. 30, 81675 Munich, Germany; Department of Pharmacy, Pharmaceutical Technology and Biopharmaceutics, Ludwig-Maximilians-Universität München, Butenandtstraße 5, 81377 Munich, Germany; Institute of Virology, School of Medicine, Technische Universität München / Helmholtz Zentrum München, Trogerstr. 30, 81675 Munich, Germany; Institute of Lung Biology and Disease (ILBD) and Comprehensive Pneumology Center (CPC) with the CPC-M bioArchive, Helmholtz Zentrum München, Member of the German Center for Lung Research (DZL), Munich, Germany; Institute of Lung Biology and Disease (ILBD) and Comprehensive Pneumology Center (CPC) with the CPC-M bioArchive, Helmholtz Zentrum München, Member of the German Center for Lung Research (DZL), Munich, Germany; Institute of Virology, School of Medicine, Technische Universität München / Helmholtz Zentrum München, Trogerstr. 30, 81675 Munich, Germany; Department of Medicine V, University Hospital, LMU Munich, Member of the German Center for Lung Research (DZL), Munich, Germany; Center for Thoracic Surgery Munich, Ludwig-Maximilians-University of Munich (LMU) and Asklepios Pulmonary Hospital; Marchioninistraße 15, 81377 Munich and Robert-Koch-Allee 2, 82131 Gauting, Germany; Institute of Virology, School of Medicine, Technische Universität München / Helmholtz Zentrum München, Trogerstr. 30, 81675 Munich, Germany; Institute of Lung Biology and Disease (ILBD) and Comprehensive Pneumology Center (CPC) with the CPC-M bioArchive, Helmholtz Zentrum München, Member of the German Center for Lung Research (DZL), Munich, Germany; Institute of Virology, School of Medicine, Technische Universität München / Helmholtz Zentrum München, Trogerstr. 30, 81675 Munich, Germany; German Center for Infection Research (DZIF), Munich partner site, Germany; Department of Pharmacy, Pharmaceutical Technology and Biopharmaceutics, Ludwig-Maximilians-Universität München, Butenandtstraße 5, 81377 Munich, Germany; Institute of Lung Biology and Disease (ILBD) and Comprehensive Pneumology Center (CPC) with the CPC-M bioArchive, Helmholtz Zentrum München, Member of the German Center for Lung Research (DZL), Munich, Germany; Institute of Virology, School of Medicine, Technische Universität München / Helmholtz Zentrum München, Trogerstr. 30, 81675 Munich, Germany; Infectious Diseases Therapeutic Research Center, Therapeutics & Biotechnology Division, Korea Research Institute of Chemical Technology (KRICT), 34114 Daejeon, Republic of Korea; Institute of Virology, School of Medicine, Technische Universität München / Helmholtz Zentrum München, Trogerstr. 30, 81675 Munich, Germany; German Center for Infection Research (DZIF), Munich partner site, Germany

## Abstract

A promising approach to tackle the severe acute respiratory syndrome coronavirus-2 (SARS-CoV-2) could be small interfering (si)RNAs. So far it is unclear, which viral replication steps can be efficiently inhibited with siRNAs. Here, we report that siRNAs can target genomic RNA (gRNA) of SARS-CoV-2 after cell entry, and thereby terminate replication before start of transcription and prevent virus-induced cell death. Coronaviruses replicate via negative sense RNA intermediates using a unique discontinuous transcription process. As a result, each viral RNA contains identical sequences at the 5′ and 3′ end. Surprisingly, siRNAs were not active against intermediate negative sense transcripts. Targeting common sequences shared by all viral transcripts allowed simultaneous suppression of gRNA and subgenomic (sg)RNAs by a single siRNA. The most effective suppression of viral replication and spread, however, was achieved by siRNAs that targeted open reading frame 1 (ORF1) which only exists in gRNA. In contrast, siRNAs that targeted the common regions of transcripts were outcompeted by the highly abundant sgRNAs leading to an impaired antiviral efficacy. Verifying the translational relevance of these findings, we show that a chemically modified siRNA that targets a highly conserved region of ORF1, inhibited SARS-CoV-2 replication *ex vivo* in explants of the human lung. Our work encourages the development of siRNA-based therapies for COVID-19 and suggests that early therapy start, or prophylactic application, together with specifically targeting gRNA, might be key for high antiviral efficacy.

## Graphical Abstract

**Graphical Abstract:**
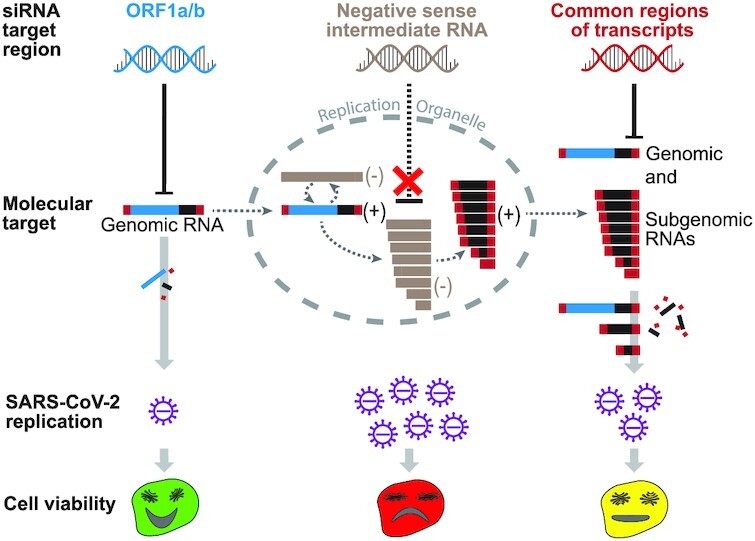
Exclusive targeting of SARS-CoV-2 genomic RNA enables most efficient suppression of viral replication and virus-induced cell death.

## INTRODUCTION

Severe acute respiratory syndrome coronavirus-2 (SARS-CoV-2) is causing a pandemic with disastrous consequences on global health, politics and economy. SARS-CoV-2, like other coronaviruses affecting humans, is mainly transmitted via respiratory secretions (1), and replicates primarily in respiratory epithelial cells (2). Due to its lytic cell cycle (3), it causes severe endothelial injury and widespread microangiopathy (4), which can trigger a pathological cascade that can lead to respiratory failure and death (5). While some progress has been made by repurposing the RNA polymerase inhibitor Remdesivir (6), using monoclonal antibodies against the receptor-binding domain of the viral Spike (S) protein (7), or by ameliorating SARS-CoV-2 induced lung injury using dexamethasone (8), the impact of such therapies on lethality of coronavirus disease 19 (COVID-19) remains limited (9). Several potential new treatments are currently investigated (10). One promising approach could be to deliver small interfering (si)RNAs locally to the respiratory tract by inhalation (11), and induce degradation of viral RNAs by the RNA interference (RNAi) machinery. Studies performed with severe acute respiratory syndrome coronavirus-1 (SARS-CoV-1) or Middle East respiratory syndrome coronavirus (MERS-CoV), showed that siRNAs can silence viral RNA and relieve symptoms caused by related coronaviruses (12–15). The ongoing pandemic prompted multiple research groups to evaluate siRNA-based therapies for COVID-19. While most of the so far published studies reviewed the potential of RNAi to treat COVID-19 (16–21), describe in-silico studies (22–28), or are restricted to using reporter assays to test activity of siRNAs (29,30), initial proof-of-concept that SARS-CoV-2 can be inhibited by siRNAs, was also provided (31,32). However, until today it is unclear, which viral replication steps are accessible for RNAi and which are the determinants for an efficient suppression of viral replication. An in-depth understanding of these factors, however, would be a requirement to formulate a potent antiviral strategy.

SARS-CoV-2, as other coronaviruses, has a positive sense, single-stranded RNA genome with a length of ∼30 000 nucleotides. Following binding to the cellular receptor angiotensin-converting enzyme 2 (33), the virus is taken up via endocytosis (34). After fusion with the endosomal membrane with the help of the host protease transmembrane protease serine 2 (35), the ribonucleocapsid is released into the cytoplasm. Here, the viral genome serves as template for translation of the polyprotein 1ab (pp1ab) from open reading frame 1 (ORF1) by the cellular ribosomal machinery. Pp1ab is cleaved into 16 non-structural proteins (NSPs) of which several assemble around the viral genome to form the replication/transcription complex (RTC) (36). As for other positive sense RNA viruses, transcription does not take place in the cytosol, but exclusively within double-membrane vesicles (37). Therefore, the viral RTC associates with endoplasmic reticulum membranes to form viral replication organelles (ROs). Here, the viral genome serves as template for transcription of full-length progenitor genomic (g)RNA as well as subgenomic (sg)RNAs encoding for structural (S, envelope protein [E], membrane protein [M], Nucleocapsid [N]) as well as accessory proteins (3a, 6, 7a, 7b, 8 and 10) (38). Replication takes place via negative sense intermediate RNAs in a process called discontinuous transcription (39,40). As a result, each coronaviral RNA contains an identical 5′ (the ∼70 nucleotide long leader sequence [L]) as well as 3′ end (N ORF and 3′ untranslated region [3′UTR]) (38). Next, sgRNAs are released from ROs (41), translated into the corresponding protein and gRNA packaged by the structural proteins to assemble progeny virions.

Coronaviruses protect their RNA well. Besides the lipid bilayer envelope, nucleocapsid proteins bind directly to the viral genome. Thus, even between uncoating and incorporation into double-membraned ROs, the genome is not present as naked RNA (42). Furthermore, while sgRNAs are exported from ROs for translation, this does not seem to be the case for gRNA which remains associated with double-membraned vesicles (41). Currently, it is not clear whether and how the different viral RNA species can be targeted by an RNAi-based therapy. Furthermore, certain viral components might be essential for replication, whereas the loss of others might be tolerated by the virus. Thus, suppression of reporter constructs as often performed during siRNA development may not accurately predict the effect of siRNAs on viral replication and spread. To shed light upon these questions, we systematically analyzed which viral RNA species and steps of the SARS-CoV-2 life cycle can be targeted by siRNAs and how this would affect viral replication.

## MATERIALS AND METHODS

### siRNA design and synthesis

**Figure 1.**
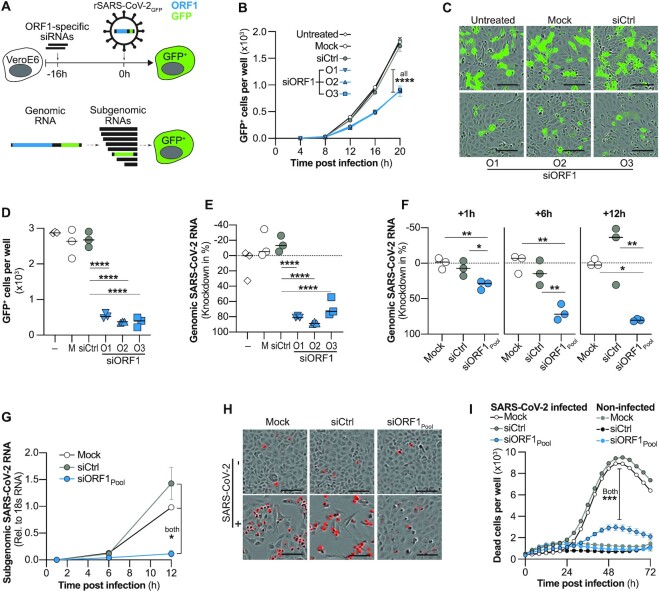
Effect of targeting genomic SARS-CoV-2 RNA with siRNAs on viral replication and cytopathy. (**A**, top) Experimental setup used in (B–D). VeroE6 cells were transfected with siRNAs targeting ORF1 (siORF1) 16h before infection with recombinant, GFP-expressing SARS-CoV-2 (rSARS-CoV-2-GFP; MOI 1) and number of GFP^+^ positive cells quantified. Cells receiving no treatment (untreated), transfection reagent only (Mock) or a control siRNA (siCtrl) served as controls. (A, bottom) Schematic representation of gRNA, as well as sgRNAs. Note that ORF1 (blue) is only part of full-length gRNA but not sgRNAs. GFP, green fluorescent protein. (**B**) Kinetic of viral spread showing number of GFP^+^ cells determined by automated quantification using the integrated Incucyte S3 software. (C, D) GFP expression 24h after infection with rSARS-CoV-2-GFP. (**C**) Exemplary fluorescence microscopy pictures. Bar at lower right indicates 0.1 mm length and (**D**) quantification of GFP^+^ cells. (**E**) Same experimental setup as in (B–D) but cells were infected with *wildtype* SARS-CoV-2 (MOI 0.1) and lysed after 24 h to quantify genomic SARS-CoV-2 RNA from cell lysate by RT-qPCR. (F, G) siRNAs used in (B–E) were pooled and transfected into VeroE6 cells 6h before infection with *wildtype* SARS-CoV-2. Cells were lysed at different time points after infection and SARS-CoV-2 (**F**) gRNA as well as (**G**) sgRNAs quantified by RT-qPCR. (H, I) VeroE6 cells were transfected with siRNAs 6h before infection with *wildtype* SARS-CoV-2 (MOI 1) and dead cells visualized using the Incucyte® Cytotox Red Dye and quantified using the Incucyte S3. (**H**) Exemplary fluorescent microscopy pictures taken at 56h p.i. Dead cells are shown in red. Bar at lower right indicates 100 μm length. (**I**) Time kinetic of dead cells quantified every 4h over a period of 3 days. (B, G, I) Mean of triplicates for each treatment group is shown, error bars indicate SEM. Bars in (D–F) show median. Statistical differences were calculated using (B, G, I) repeated measures one-way Anova or (D–F) regular one-way Anova with Dunnett′s multiple comparison correction. M, Mock; –, untreated; O1-3, ORF1-specific siRNAs 1–3; *P < 0.05; **P < 0.01; ***P < 0.001; ****P < 0.0001.

**Figure 2.**
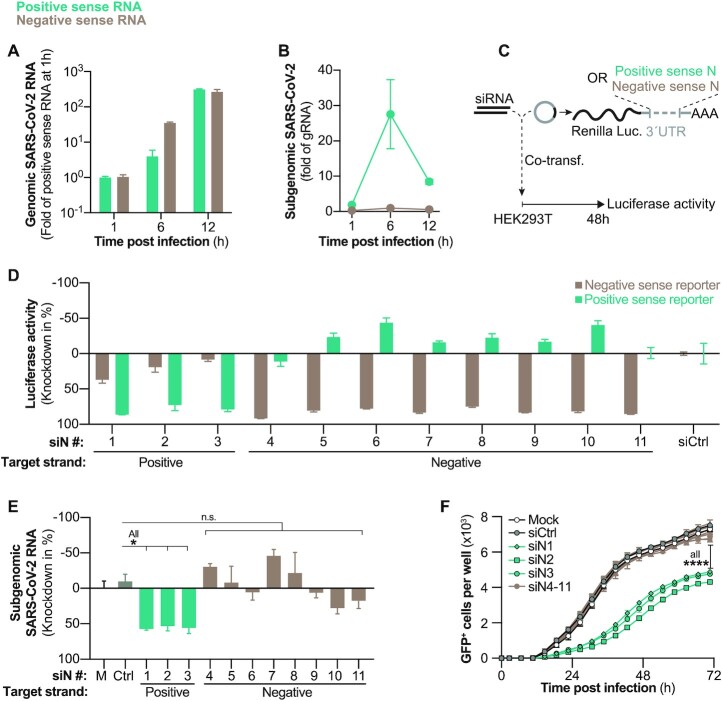
Evaluation of SARS-CoV-2 negative sense RNA as siRNA target. (A, B) Kinetics of negative and positive sense SARS-CoV-2 RNAs following *wildtype* SARS-CoV-2 infection (MOI 0.1) of VeroE6 cells. Negative and positive sense RNAs were individually transcribed to cDNA by using either poly A or poly T primers and (**A**) gRNA and (**B**) sgRNAs quantified by RT-qPCR. (**C**) Experimental setup to determine siRNA strand specific activities. Luciferase reporters with incorporated positive or negative sense N sequences in the 3′UTR of R*enilla* luciferase were co-transfected with siRNAs into HEK293T cells and (**D**) luciferase activity measured after 48 h (**E**) siRNAs were transfected into VeroE6 cells 6 h before infection with *wildtype* SARS-CoV-2 (MOI 0.1) and 24 h p.i. sgRNAs quantified from cell lysate using RT-qPCR. (**F**) Same setup as in (E) but VeroE6 cells were infected with rSARS-CoV-2-GFP (MOI 1.0) and GFP^+^ cells quantified every 4 h. All experiments were performed with three biological replicates. Graphs in (A, B, D, E) show mean and error bars SEM. Statistical differences were calculated using (E) Regular or (F) repeated measures one-way Anova with Dunnett′s multiple comparison correction. Co-transf., co-transfection; M = mock-transfected; n.s., non-significant, *P < 0.05; ****P < 0.0001

**Figure 3.**
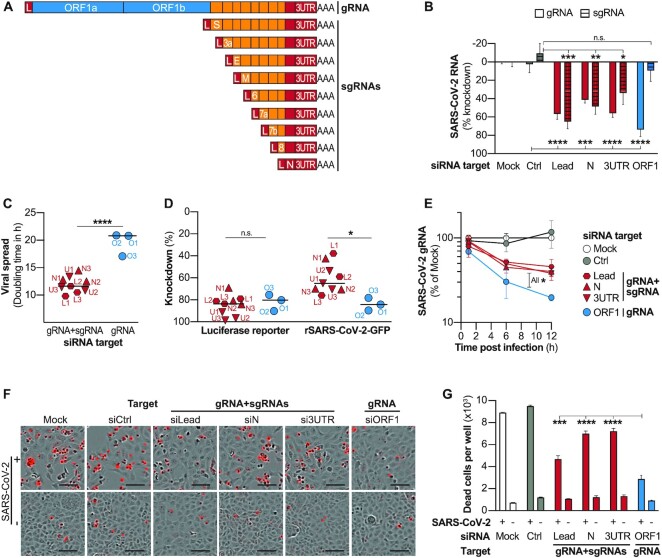
Targeting common regions of SARS-CoV-2 transcripts allows simultaneous suppression of gRNA and sgRNAs, but leads to impaired antiviral activity. (**A**) Schematic presentation of SARS-CoV-2 transcripts with sequences that are found in several transcripts shown in orange or red, and sequences that are exclusively part of viral gRNA shown in blue. (**B**) Effect of siRNAs targeting ORF1 which is only part of full-length SARS-CoV-2 gRNA or targeting sequences common within gRNA and sgRNAs. VeroE6 cells were infected with *wildtype* SARS-CoV-2 (MOI 0.1) and 3 h p.i. transfected with siRNA pools (containing three siRNAs each) specific for indicated genomic regions of SARS-CoV-2. At 24 h p.i., viral gRNA and sgRNAs were quantified by RT-qPCR. gRNA levels are shown relative to 18S rRNA and sgRNA relative to gRNA. (**C**) VeroE6 cells were infected with rSARS-CoV-2-GFP (MOI 1) and 3 h later transfected with individual siRNAs targeting indicated genomic regions of SARS-CoV-2. GFP^+^ cells were quantified every 4h (for full data see Supplementary Figure S3C) and virus spread quantified by fitting an exponential curve and calculating the doubling time. Dots represent median of three biological replicates each. Name of siRNA is given by red and blue labeling; L1–3; Leader-sequence specific siRNAs 1–3; N1–3, N-specific siRNAs 1–3; U, 3′UTR-specific siRNAs 1–3; O1–3, ORF1-specific siRNAs 1–3. (**D**) Comparison of siRNA efficacy against luciferase reporters or SARS-CoV-2 infection. To determine activity against luciferase reporters, each siRNA was transfected together with the respective luciferase reporter into HEK293T cells and luciferase activity measure after 48 h. To measure antiviral activity, experimental setup as described under (C) was used, and GFP^+^ cells quantified at final time point (68 h). Each dot represents median of three biological replicates. (**E**) VeroE6 cells were transfected with siRNA pools and infected with *wildtype* SARS-CoV-2 (MOI 0.1) after 6 h. Viral gRNA was quantified relative to 18srRNA at given time points using RT-qPCR (F, G). Effect of siRNA treatment on SARS-CoV-2 induced cytolysis. VeroE6 cells were transfected with siRNA pools and infected with *wildtype* SARS-CoV-2 (MOI 1) after 6 h. Virus-induced cell death was analysed using the Incucyte^®^ Cytotox Red Dye at 56 h p.i. (**F**) Exemplary fluorescence microscope images showing dead cells in red. Bars in lower right of images represent 100 μm. (**G**) Number of dead cells were quantified using the Incucyte S3 analyzing system. Bar in (C, D) shows median. (B, E, G) show mean ± SEM. Statistical differences were calculated using (**B, G**) one-way Anova, or (**E**) repeated measures Anova with Dunette′s multiple comparison correction and in (C, D) using Student's *t*-test for independent samples. All experiments were performed using three biological replicates. n.s., non-significant; *P < 0.05; **P < 0.01; ***P < 0.001; ****P < 0.0001.

**Figure 4.**
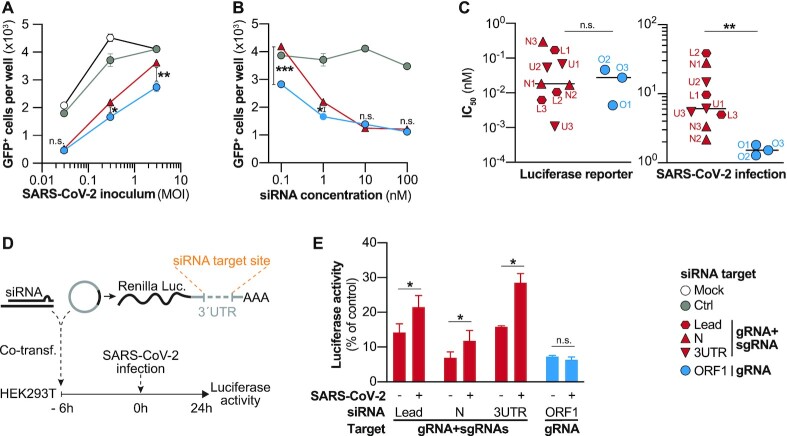
Subgenomic RNAs out-compete and impair antiviral activity of siRNAs. (A, B) VeroE6 cells were transfected with siRNAs targeting sgRNA and gRNA (N2) or exclusively gRNA (O2), infected 6h later with rSARS-CoV-2-GFP and the number of GFP^+^ cells was determined 24 h p.i. (**A**) siRNAs were transfected at a concentration of 1 nM, and cells were infected with MOIs of 0.03, 0.3 and 3. (**B**) siRNAs were transfected at varying concentrations ranging from 0.1 to 100 nM and VeroE6 were infected with a MOI of 0.3. (**C**) Comparison of mean inhibitory concentrations (IC_50_) of siRNAs determined using luciferase reporters (left graph) or rSARS-CoV-2-GFP (right graph). Full data is shown in Supplementary Figures S4 and S5. For experimental details see Materials and Methods section. (D, E) Competition experiment to determine effect of SARS-CoV-2 replication on RNAi silencing efficacy. (**D**) HEK293T cells were co-transfected with siRNAs against different target region as well as luciferase reporters with incorporated binding sites for the co-transfected siRNA. After 6h, cells were infected with *wildtype* SARS-CoV-2 (MOI 0.1) and (**E**) luciferase activity determined from cell lysate 24 h p.i.. Statistical differences were calculated using Student's *t*-test for independent samples; n.s., non-significant, *P < 0.05; **P < 0.01.

We designed siRNAs against the SARS-CoV-2 Leader sequence, ORF1, Nucleocapsid gene (N) and 3′ untranslated region (3′UTR) employing a publicly available online tools (43) using the full-length reference sequence (NCBI Accession number: NC_045512.2) from the RefSeq database as a template. For a fair comparison of target regions, siRNAs for which a similar silencing efficacy was predicted (44) were further incorporated in the study. The siRNAs were designed in two versions: (i) As symmetric siRNAs with a length of 21 or 23 nucleotides with 2 nucleotide overhangs at the 3′ ends of both strands and occasional G:U wobbles at the 5′ end of the antisense strand (45) to improve specificity (43) (Table 1 and Supplementary Table S1; experiments shown in Figures 1–4 and Supplementary Figures S1–S5). (ii) To exclude a bias by the slight variations in the siRNA design (different lengths and containment of wobbles), we additionally ordered siRNAs against the same target sites that all had an identical design (symmetric 21-mers with 2 nucleotide overhangs at 3′ ends of both strands [sense strand overhang consisting of dTdT] and no wobbles) (Supplementary Table S3; experiments shown in Supplementary Figure S6). Two additional siRNAs targeting GFP (siGFP) and Firefly Luciferase (siLuc) were designed as negative controls. siLuc served as control for SARS-CoV-2 infection experiments and siGFP for experiments in which Luciferase reporters were used. All chemically unmodified siRNAs were purchased in desalted form (Microsynth AG, Balgach, Switzerland), resuspended and maintained in RNAse free water upon arrival.

**Table 1.** Sequences of siRNAs used in the study. siRNA duplexes were designed with occasional G:U wobbles at the 5′ end of the antisense strand, as indicated by small lettered ‘u’. L1–3; leader-sequence specific siRNAs 1-3; O1–3, ORF1-specific siRNAs 1–3; N1–3, N-specific siRNAs 1–3; U, 3′UTR-specific siRNAs 1–3; GFP = Green Fluorescent Protein; Luc = Firefly Luciferase; A = adenine; C = cytosine; G = guanine; U = uracil; T = thymine

Name	Sense strand (5′-3′)	Antisense strand (5′-3′)
L1	UCUGUUCUCUAAACGAAuUTT	AGUUCGUUUAGAGAACAGAUC
L2	CCAACCAACUUUCGAUuUuTT	GAGAUCGAAAGUUGGUUGGUU
L3	AAACCAACCAACUUUCGAUTT	AUCGAAAGUUGGUUGGUUUGU
O1	CCAAAUGUGCCUUUCAACUTT	AGUUGAAAGGCACAUUUGGUU
O2	GUUACAUGCACCAUAUGGATT	UCCAUAUGGUGCAUGUAACAA
O3	GGUACUUGGUAGUUUAGCUTT	AGCUAAACUACCAAGUACCAU
N1	GAAUAAGCAUAUUGACGuATT	UGCGUCAAUAUGCUUAUUCAG
N2	CAAAUUGGCUACUACCGAATT	UUCGGUAGUAGCCAAUUUGGU
N3	CGCUUCAGCGUUCUUCGGAAUTT	AUUCCGAAGAACGCUGAAGCGTT
N4	GGACGAUUGUUACGACGUUTT	AACGUCGUAACAAUCGUCCUA
N5	CCCUUGAAGAGGACGAUuUTT	AGAUCGUCCUCUUCAAGGGGA
N6	CGUGGGCGUUAGGACGAUUTT	AAUCGUCCUAACGCCCACGGU
N7	GAUUGUUUCUGCCGUAGUATT	UACUACGGCAGAAACAAUCGU
N8	GGGUGGUUGUCUCGGAUUUTT	AAAUCCGAGACAACCACCCUU
N9	GUUCCUUGUUGUAACGGUUTT	AACCGUUACAACAAGGAACUC
N10	GACGAUUGUUACGACGUUATT	UAACGUCGUAACAAUCGUCCU
N11	CUAGUUCAGUAAAACGAuUTT	AGUCGUUUUACUGAACUAGAA
U1	CUUUAAUCAGUGUGUAACATT	UGUUACACACUGAUUAAAGAU
U2	CCUAAUGUGUAAAAUUAAUTT	AUUAAUUUUACACAUUAGGGC
U3	CAUGUGAUUUUAAUAGCUUTT	AAGCUAUUAAAAUCACAUGGG
siGFP	GCAGCACGACUUCUUCAAGTT	CUUGAAGAAGUCGUGCUGCTT
siLuc	CGUACGCGGAAUACUUCGATT	UCGAAGUAUUCCGCGUACG

Chemically modified versions of ORF1-targeting siRNAs and siLuc were designed in an asymmetric fashion using a previously described design and chemical modification pattern (46) as employed for Lumasiran (47). In brief, all nucleotides of the siRNA were subjected to a 2′-*O*-methyl modification (2′OMe) except nucleotides at positions 7, and 9–11 of the siRNA sense-, as well as positions 2, 6, 8, 9, 14 and 16 of the antisense-strand (all 5′-3′ direction), which contained 2′-Fluoro modifications (2′F) instead. Additionally, two consecutive nucleotides at both ends of the siRNA antisense strand, as well as at the 5′ end of the sense strand were incorporated with phosphorothioate linkages (for details see Supplementary Table S4). Chemically modified siRNAs were synthesized by Eurogentec (Liège, Belgium) at a 40 nmol scale and purified by high performance liquid chromatography (HPLC). The siRNAs were obtained in desalted form and reconstituted in RNAse free water at a concentration of 20 mM.

### Conservation of siRNA target sites

To analyze the conservation of the siRNA target sites within the global SARS-CoV-2 population, we downloaded (date of retrieval: 26 October 2021) SARS-CoV-2 sequences from the GISAID EpiCoV™ Database (48) using the most stringent quality indicators (only complete sequences with high sequencing coverage). To analyze the conservation of siRNA target sites within currently circulating SARS-CoV-2 strains without bias, we retrieved the 100 000 sequences with latest submission date (ranging from 1 October until 26 October 2021) without restriction to a specific lineage. For a more in-depth analysis of the SARS-CoV-2 variants that were defined by the WHO by the day of sequence retrieval as major variants of concern (VoC) or interest (VoI), we downloaded 20 000 sequences (each time considering only the latest submission dates) of each of the four VoC (WHO labels: Alpha, Beta, Gamma, and Delta variant) and all available sequences for the two VoI (WHO labels: Lambda and Mu variant) for which less sequences were available (848 and 5889). These included lineages that were defined by the Pango nomenclature system (27) as: B.1.1.7 and Q.x (Alpha variant); B.1.351, B.1.351.2 and B.1.351.3 (Beta variant), P.1 and P.1.x (Gamma variant), B.1.617.2 and AY.x (Delta variant), C.37 and C.37.1 (Lambda variant), as well as B.1.621 and B.1.621.1 (Mu variant). For each of the 12 siRNAs, a search was performed for the presence of the siRNA target site within each of the acquired data sets using an inhouse script written in Ruby programming language (https://www.ruby-lang.org). Only perfect matches were counted, and the fraction of SARS-CoV-2 sequences containing the match presented in percent.

### Prediction of secondary structure of siRNA target sites

The stability of RNA secondary structures of regions that were targeted by our siRNAs were analyzed by making use of data provided by Andrews et al. (49) who analyzed all possible 120-nucleotide windows (each shifted by 1 nucleotide) of the SARS-CoV-2 genome using the ‘ScanFold’ algorithm (50). We averaged the values of four consecutive 120-nucleotide windows that contained the respective siRNA target site in the center to calculate the mean ‘native dG score’ (or ‘minimum free energy’ [MFE]), the thermodynamic ‘z-score’, and the GC content for each region. The native dG score predicts the free energy value of the most stable possible structure the sequence could adopt. A more negative value represents a more stable structure, corelating with less efficient RNAi activity (51). The z-score refers to the difference of minimum free energy between a potentially folded structural RNA and a random RNA of the same dinucleotide frequency. Negative z-score indicates a window which generates a more stable structure than the sequence content would typically produce; on the contrary, positive z-score represents a less stable structure (52). The GC content positively correlates with stable secondary structures and in contrast to the other two parameters inversely with RNAi target site accessibility (53).

### Cell lines and seeding

HEK293T cells were maintained in glucose-containing Dulbecco's modified Eagle's medium (DMEM), supplemented with 10% fetal bovine serum (FBS), 2 mM l-glutamine, 50 U/ml penicillin/streptomycin, 1% non-essential amino acids and 1 mM sodium pyruvate (Gibco™-Thermo Fisher Scientific GmbH; Dreieich, Germany). VeroE6 cells were maintained in glucose containing DMEM supplemented with 5% FBS. Mycoplasma contaminations were excluded in all cell lines. Cells were kept at 37°C in humidified incubators at 5% CO_2_. 200 000 HEK293T cells were plated in poly-l-lysine (Sigma-Aldrich Chemie; Taufkirchen, Germany) treated 24-well plates for reporter assays, 150 000 or 20 000 VeroE6 cells were plated in 24-well or 96-well plates (Techno Plastic Products; Trasadingen, Switzerland) respectively for experiments including SARS-CoV-2 infection.

### Human tissue, ethics statement and human precision-cut lung slices (hPCLS)

Human tissue was obtained from the CPC-M bioArchive at the Comprehensive Pneumology Center (CPC), from the University Hospital Großhadern of the Ludwig Maximilian University (Munich, Germany) and from the Asklepios Biobank of Lung Diseases (Gauting, Germany). Participants provided written informed consent to participate in this study, in accordance with approval by the local ethics committee of the Ludwig Maximilian University Munich, Germany (Project 19-630). PCLS were prepared as described before (54,55). Shortly, PCLS were prepared from tumor-free peri-tumor tissue. The lung tissue was inflated with 3% agarose solution and solidified at 4°C. Tissue blocks were cut in 500 μm thick slices using a vibration microtome Hyrax V50 (Karl Zeiss AG, Oberkochen, Germany). PCLS were cultured in DMEM F-12 medium supplemented with 0.1% FBS (Thermo Fisher Scientific; Dreieich, Germany). Prior to experiments, PCLS punches of 4 mm in diameter were generated using a 4 mm biopsy puncher.

### Cloning of luciferase reporters

Initial siRNA screenings, testing of siRNA strand-specific activities and the competition assay (shown in Figure 4D, E) were performed using the dual luciferase expressing psiCHECK™-2 vector (Promega GmbH; Walldorf, Germany). The siRNA target sites were cloned into a multiple cloning site present downstream of the Renilla luciferase translational stop codon via XhoI/NotI digestion (FastDigest™, Thermo Fisher Scientific; Dreieich, Germany). The binding sites of siRNAs were purchased as single-stranded DNA oligonucleotides, designed to form overhangs mimicking digested oligonucleotide fragments after annealing. Hence, equal amounts of complementary oligonucleotides were mixed and heated at 95°C for five minutes followed by gradual cooling for 2 h at 30°C to allow forming of oligonucleotide duplexes. These were directly used in a ligation reaction with the digested psiCHECK-2™ vector.

To determine strand specific siRNA activities shown in Figure 2 and Supplementary Figure S2, the full-length positive or negative sense N coding sequences were cloned into the luciferase vector. Hence, the positive sense N coding sequence was PCR amplified using primers E-N Fw BamHI and E-N Rev EcoRI from cDNA of SARS-CoV-2 infected VeroE6 cells and cloned into the pcDNA1/Amp plasmid vector. In a next step, the N-coding sequence was PCR-amplified using primers N CDS Fw XhoI and N CDS Rev NotI and cloned into the luciferase reporter. The full-length negative sense N gene was purchased as desalted, pre-annealed double-stranded DNA oligonucleotide (Eurogentec, Liège, Belgium) and used directly for the annealing reaction with digested psiCHECK™-2 vector. A list of used oligonucleotides is given in Supplementary Table S2.

### Transfection

siRNAs were transfected using Lipofectamine RNAiMAX (Thermo Fisher Scientific; Dreieich, Germany) according to manufacturer′s instructions at time points and concentrations provided in the figure legends of respective experiments. For transfections before SARS-CoV-2 infection, a reverse-transfection protocol was used. All transfection experiments were performed with at least three biological replicates. For the pre-selection of siRNAs, the determination of strand-specific activities of N-targeting siRNAs, and the competition assay, siRNAs were co-transfected together with respective plasmid expressing a luciferase reporter. In brief, 200 ng of reporter plasmid and 6 pmol of siRNA were mixed with 1 μl of transfection reagent (Lipofectamine 2000, Thermo Fisher Scientific; Dreieich, Germany) diluted with Opti-MEM to a final volume of 100 μl. siRNA and plasmid containing transfection complexes were added on top of confluent cells, resulting in 10 nM final concentration of siRNA per well. For the pre-screening of siRNAs and the determination of strand specific activities of N-specific siRNAs, constructs were transfected into 85–90% confluent HEK293T cells and for the competition assay into confluent VeroE6 cells.

Polymer/siRNA polyplexes for *ex vivo* lung transfections were prepared as described before (56) by first dissolving polyethylenimine 25 kDa (BASF, Ludwigshafen, Germany) in water at a concentration of 1 mg/ml, which was then filtered through a 0.22 μm filter for sterilization. Stocks of siRNA and PEI were further diluted in a sterile 5% glucose solution to reach the desired concentration. Polyplexes were prepared with a total amount of 60 pmol of siRNA. The required amount of PEI in μg (mPEI) was calculated as follows:

$$\begin{equation*}m{\rm PEI} = \frac{{m\left( {{\rm siRNA}} \right)}}{{M\left( {{\rm siRNA}} \right)}} \times 43.1\,{\rm g}\;{{\rm mol}^{ - 1}} \times {\rm N}/{\rm P}\end{equation*}$$

where 43.1 if the molecular weight of the protonable unit of PEI, and N/P is the ratio of protonable amines of the polymer to phosphate groups of the siRNA backbone (56). The experiment was performed at an N/P ratio of 6. A defined volume of the polymer solution was added to an equal volume of the diluted siRNA and incubated for 20 minutes at room temperature to allow polyplex formation.

### Dual-luciferase based reporter assay and competition experiment

To determine silencing activity of siRNA sequences, siRNAs were co-transfected into cells with plasmids expressing dual luciferase reporters. After co-transfecting siRNAs and plasmids (for details see paragraph above), cells were lysed after 48h (siRNA prescreening and strand specific activities) with 100 μl passive lysis buffer (Promega GmbH; Walldorf, Germany), and luciferase activity from 10 μl cell lysate measured using the Dual Luciferase^®^ Reporter Assay System (Promega GmbH; Walldorf, Germany) according to instructions using a Tecan Infinite 200 PRO Microplate reader (Tecan Group Ltd.; Männedorf, Switzerland). Relative activity of *Renilla* luciferase (normalized to Firefly luciferase activity as an internal transfection control) was indicated as silencing efficiency of the siRNA and compared to the same luciferase reporter co-transfected with the control siRNA siGFP. For the competition experiment (shown in Figure 4D, E), siRNAs and the respective luciferase reporter plasmid were co-transfected into VeroE6 cells as described previously, which were 6 h later infected with *wildtype* SARS-CoV-2 (MOI 0.1), and 24 h later, luciferase activity and knockdown efficacy were determined.

### SARS-CoV-2 infection

VeroE6 cells were seeded in 24-well format at least 6h before infection to gain ∼90–95% confluency at time of inoculation. The SARS-CoV-2 stock was pre-diluted in 200 μl growth media to achieve the desired multiplicity of infection (MOI) for the respective experiment. At time of inoculation, old growth media was removed, and the pre-diluted SARS-CoV-2 solution added to cells. After 1h incubation at 37°C, a medium exchange was performed. Experiments with *wildtype* SARS-CoV-2 were terminated at different time points ranging from 1 to 24 h post infection depending on which step of the viral replication cycle was investigated. The SARS-CoV-2 *wildtype* virus used in this study was isolated in March 2020 from a patient at the Institute of Virology, TU Munich. The full-length sequence was uploaded onto GISAID database (https://www.gisaid.org/) under name *hCoV-19/Germany/BAV-PL-virotum-nacq/2020* and accession ID: EPI_ISL_582134.

PCLS samples were prepared as described above and cultured with Dulbecco's Modified Eagle Medium (DMEM) F-12 supplemented with L-Glutamine, HEPES, 10,000 IE Penicillin, 10 000 IE streptomycin and 0.1% fetal bovine serum. For each biological replicate, three PCLS were placed in a 48-well plate in 500 μl medium and transfected with 60 pmol siRNA and PEI at N/P 6 (for details see ‘transfection’ section) six hours before being infected with wildtype SARS-CoV-2. For infection, 300 000 plague-forming units (PFU) SARS-CoV-2 were added to each well, which contained PCLS with an estimated cell number of 300 000 cells, resulting in an approximated MOI of 1.0.

### Real-time monitoring of virus spread using rSARS-CoV-2-GFP and automated fluorescence analysis with the IncuCyte® Live-Cell Analysis

VeroE6 cells in growth media were seeded at least 6h before infection into 96-well plates to gain ∼90–95% confluency at time of infection. Cell were then inoculated with a recombinant SARS-CoV-2, expressing green fluorescent protein (GFP) from a sequence integrated at the ORF7 locus (rSARS-CoV-2-GFP). For this, the rSARS-CoV-2-GFP virus infection solution was pre-diluted in 50 μl growth media to achieve the desired MOI. After adding 50 μl of the infection solution to cells, media was exchanged after 1 h, and multi-well plates placed into IncuCyte^®^ Live-Cell Analysis device for acquisition of phase contrast as well as fluorescence pictures of the entire well every 4 h for three days. Infected cell population was quantified using the GFP channel and the IncuCyte S3 software (Essen Bioscience; version 2019B Rev2).

### Half maximal inhibitory concentration

Efficacy of siRNAs to inhibit luciferase reporters or SARS-CoV-2 replication was analyzed by determining half maximal inhibitory concentrations (IC_50_). To investigate activity to suppress viral replication, siRNAs were reversely transfected into VeroE6 cells at a series of concentrations of 100, 25, 6.25, 1.56, 0.39, 0.098, 0.024 and 0.006 nM. The cells were infected with rSARS-CoV-2-GFP (MOI 1) after 6 h as described earlier. The siRNA silencing activity was determined as number of GFP^+^ cells 24 h p.i. using the Incucyte® software. To determine IC_50_ values for luciferase reporters, siRNAs were co-transfected into HEK293T cells with respective dual luciferase reporters at identical siRNA concentrations as described above and activity of firefly and Renilla luciferases measured after 48 h (for details see paragraph ‘Dual-Luciferase based reporter assay and competition experiment’). All experiments were performed using three biological replicates. IC_50_ values were calculated by fitting a nonlinear curve with variable slope using the nonlinear regression model in GraphPad 9.0 software.

### Determination of cell death and cell viability

To evaluate the impact of siRNA-treatment on SARS-CoV-2-induced cytopathy, VeroE6 cells were reversely transfected in 96-well plate with siRNAs and 6 h later infected with *wildtype* SARS-CoV-2 (MOI 1). Number of dead cells was quantified using the Incucyte^®^ Cytotox Red Dye to monitor the loss of the cell membrane integrity (Sartorius AG, Göttingen, Germany; Cat. No. 4632). As the cyanine nucleic acid dye is unable to pass the plasma membranes of healthy cells, the dye can only bind to DNA if the integrity of cellular membranes is compromised. Fluorescence signal (maximum at 631 nM) was measured using the red channel of the Incucyte S3 analyzing system every 4 h for 3 days after infection. As a further marker of cell viability, the metabolic rate of treated cells was determined using the CellTiter-Blue Cell Viability Assay kit (Promega GmbH, Walldorf, Germany) according to manufacturer's instructions. Accordingly, CellTiter-Blue reagent was diluted 1:5 with culture medium and applied to cells for 1 h at 37°C, 5% CO_2_. Conversion from resazurin to resorufin was analyzed with fluorescence filters 550/590 nm from a Tecan Infinite F200 (Tecan Group Ltd.; Männedorf, Switzerland).

### Nucleic acid extraction and qPCR

RNA from cultured cells was extracted with the NucleoSpin RNA kit (Macherey-Nagel; Düren, Germany), and cDNA synthesized with the Superscript™ III First-Strand Synthesis System (Thermo Fisher Scientific; Dreieich, Germany) according to manufacturer's instructions. SARS-CoV-2 transcripts were amplified in subsequent qPCR using primers specific for the N region, essentially covering all the viral transcripts or the RNA dependent RNA polymerase (Rdrp) region, as a measure of gRNA. For quantification of viral RNAs, a standard curve was constructed using plasmids with integrated Rdrp or N sequences. Amount of sgRNAs was calculated by subtracting the number of Rdrp containing transcripts (as a marker of gRNA) from the N-containing transcripts as full-length gRNA is also detected by the N primers. 18S rRNA was used as a reference gene for relative quantification. All quantitative PCRs were performed on a LightCycler^®^ 480 (Roche Holding AG; Basel, Switzerland) using primers and cycling conditions shown in Table 2.

**Table 2.** Oligonucleotides and cycling conditions used during polymerase change reaction. A = adenine; C = cytosine; G = guanine; T = thymine; Rev = reverse; min = minute; s = second; RDRP = RNA-dependent RNA polymerase

Primers	Sequence (5′-3′)
N CDS Fw XhoI	ATCATACTCGAGATGTCTGATAACGGACCCCA
N CDS Rev NotI	ATCATTGCGGCCGCGGCCTGAGTTGAGTCAGCAC
E-N fw BamHI	GGTGGTGGATCCTGAGCCTGAAGAACATGTCC
E-N Rev EcoRI	GGTGGTGAATTCAGCTCTCCCTAGCATTGTTC
Oligo(dT)_20_	TTTTTTTTTTTTTTTTTTTT
Oligo(dA)_20_	AAAAAAAAAAAAAAAAAAAA
18S cDNA 1	CCTTCCGCAGGTTCACCTAC
18S cDNA 2	CCTCCAATGGATCCTCGT
18S cDNA 3	TAATCATGGCCTCAGTTCCG
18S qPCR	Fw: AAACGGCTACCACATCCA
	Rev: CCTCCAATGGATCCTCGT
N qPCR	Fw: GACCCCAAAATCAGCGAAAT
	Rev: TCTGGTTACTGCCAGTTGAATCTG
RDRP qPCR	Fw:CGTCTGCGGTATGTGGAAAG
	Rev: TAAGACGGGCTGCACTTACA
PCR cycling conditions:	Initial Denaturation: 95°C 5 Min (Ramp rate 4.4)
	45 Cycles: 95°C - 15 seconds (Ramp rate 4.4)
	55°C - 10 seconds (Ramp rate 2.2)
	72°C - 25 seconds (Ramp rate 4.4)

### Strand-specific cDNA synthesis

To individually determine negative or positive sense SARS-CoV-2 RNA, we specifically transcribed RNA of a certain polarity to cDNA. Hence, first strand synthesis was performed from total RNA extracts using the SuperScript™ IV First-Strand Synthesis System (Thermo Fisher Scientific; Dreieich, Germany) with primers specific either for positive sense mRNA (Oligo(dT)_20_ primers) or negative sense mRNA (Oligo(dA)_20_ primers). To allow transcription of a house keeping gene also in the reaction transcribing negative sense RNA, primers specific for the 18S rRNA gene (18S cDNA1-3; Table 2) were added to the reaction. A final concentration of 50 μM for all primers combined were used for first strand synthesis reaction and viral RNAs quantified by qPCR as described above.

### Statistical analysis

Statistical analysis was performed with GraphPad Prism (version 8.4.3) for Mac. Normally distributed samples were analyzed using the Student T-test for independent samples when comparing two groups and with One-way Anova with Dunnett′s multiple comparison correction when comparing three or more groups. Statistical differences of non-normally distributed data were calculated for two groups using Mann–Whitney *U* or Kruskal–Wallis with Dunn′s multiple comparison correction tests when comparing three or more groups. *P*-values <0.05 were considered significant.

## RESULTS

### Targeting the genome of SARS-CoV-2 with siRNAs terminates replication before start of transcription and prevents virus-induced cell death

Following the events in the viral replication cycle, first, we investigated whether siRNAs can directly target the incoming genome of SARS-CoV-2 after cell entry. We chose ORF1 as target region, as it is only contained in full-length genomic, but not sgRNAs. We individually transfected three siRNAs which were active in previous luciferase reporter screens (Supplementary Figure S1A) into VeroE6 cells. After 16 h, cells were infected with a recombinant SARS-CoV-2 (rSARS-CoV-2-GFP), which expresses GFP from an integrate at the ORF7 locus. Viral infection and spread were monitored by quantifying GFP^+^ cells every 4h over the course of three days (Figure 1A, top). As the ORF1-specific siRNAs do not target the transcript from which GFP is expressed, a suppression of GFP expression would indicate that siRNAs targeted full-length gRNA (Figure 1A, bottom). Indeed, we found the number of GFP^+^ cells reduced to ∼50% by each of the tested siRNAs (Figure 1B–D; Supplementary Figure S1B). Importantly, this difference was already present at the earliest time point (12 h post infection [p.i.]) with detectable GFP signal (Figure 1B), indicating that genomes of incoming virus were successfully targeted. We confirmed this by repeating the experiment using *wildtype* SARS-CoV-2 but lysed the cells 24 h p.i. and quantified SARS-CoV-2 gRNA by RT-qPCR. As indicated by our previous experiment, gRNA was reduced in groups pre-treated with the ORF1-specific siRNAs (Figure 1E). To further confirm that indeed genomes of incoming virus were degraded, we transfected cells with a pool of three ORF1-specific siRNAs 6 h before infection with *wildtype* SARS-CoV-2 and quantified intracellular viral RNAs at different time points. Viral RNAs were further differentiated into full-length gRNA and sgRNAs (see Materials and Methods for details). We found that gRNA was reduced as early as 1 h p.i. (Figure 1F), before sgRNAs were synthesized (Figure 1G). Treatment with ORF1-specific siRNAs prevented sgRNA expression (Figure 1G), improved cell viability (Supplementary Figure S1C) and prevented cell death (Figure 1H, I). Taken together, our data demonstrates that siRNAs can target the genome of SARS-CoV-2 and terminate viral replication at an early replication step and by this prevent cytopathy.

### Negative sense SARS-CoV-2 RNAs are not accessible for siRNA therapy

Currently it is unclear if both, negative and positive sense coronaviral RNA, or only RNA with a certain polarity is accessible for RNAi silencing. This question is particularly interesting when designing therapeutic siRNAs, as potentially both strands of the siRNA could convey antiviral activity. To gain a more detailed understanding on the kinetic of RNA synthesis during SARS-CoV-2 replication, we lysed *wildtype* SARS-CoV-2-infected VeroE6 cells at different time points. Positive and negative sense viral RNAs were individually quantified by strand-specific first strand synthesis (see Materials and Methods). Negative sense gRNA was detected in low quantities already 1 h p.i., but strongly increased at 6 h p.i. when it was more abundant than positive sense gRNA (Figure 2A). In contrast, sgRNAs started to appear only at 6 h p.i. (Figure 2B). Consistent with other coronaviruses, lower amounts of negative sense sgRNAs were detected as compared to their positive sense counterparts (57). We then investigated whether negative sense SARS-CoV-2 RNA is accessible for RNAi-mediated silencing. We developed siRNAs that specifically targeted either negative or positive sense SARS-CoV-2 RNA. We chose the N ORF as target region, as it is also part of sgRNAs which are – in contrast to gRNA—exported from ROs (41), and should therefore be easily accessible for siRNAs. siRNA strand-specific activity was validated by co-transfecting siRNAs with reporter plasmids that either carried the positive or negative sense N coding sequence in the 3′UTR of the *Renilla* luciferase gene (see scheme in Figure 2C and methods section). The majority of siRNAs presented a selectivity for the RNA strand they were designed against (Supplementary Figure S2). We chose siRNAs with almost exclusive activity against either the positive or negative sense reporter (Figure 2D) and tested their antiviral activity. To our surprise, only siRNAs active against positive sense N ORF reduced sgRNAs during *wildtype* SARS-CoV-2 infection (Figure 2E), and inhibited viral spread in the rSARS-CoV-2-GFP model (Figure 2F). In summary, our data proves that negative sense SARS-CoV-2 RNAs are inaccessible for RNAi.

### siRNA-targeting of the common regions of SARS-CoV-2 transcripts allows simultaneous suppression of gRNA and sgRNAs, but leads to reduced antiviral efficacy

We further went on to investigate whether targeting the common regions shared by all SARS-CoV-2 transcripts (L, N ORF and 3′UTR; see scheme in Figure 3A) would allow simultaneous suppression of gRNA as well as sgRNAs, and how this would affect antiviral efficacy. To achieve a fair comparison between target regions, we selected three siRNAs for each target region for which a similar efficacy was predicted by the design tool (Supplementary Figure S3A) and which suppressed luciferase reporters to comparable degrees (Supplementary Figure S3B), with only siRNAs against the leader sequence showing slightly lower scores, as the small size of the target limited options for siRNA design. To not interfere with incoming SARS-CoV-2 genomes of input virus, we first infected VeroE6 cells with *wildtype* SARS-CoV-2 and transfected the siRNAs 3h later. To compensate for the differences of the activities of individual siRNAs, we pooled three siRNAs for each target region and tested their effect on SARS-CoV-2 RNA expression (Figure 3B). As expected, ORF1-specific siRNAs suppressed only gRNA, whereas siRNAs targeting common regions of transcripts suppressed gRNA and sgRNAs. We next investigated how targeting sgRNAs in addition to gRNA would affect antiviral efficacy of siRNAs. To this end, we infected cells with rSARS-CoV-2-GFP and this time transfected the three siRNAs per target region individually. All siRNAs significantly inhibited viral replication and viral dissemination as evidence by lower frequency of GFP-expressing cells as compared to controls (Supplementary Figure S3C). To our surprise, however, SARS-CoV-2 spread significantly slower in groups treated with siRNAs that solely targeted gRNA (target region ORF1), illustrated by increased doubling times of GFP^+^ cells (Figure 3C). While both groups of siRNAs (targeting only gRNA or additionally sgRNAs) suppressed luciferase reporters to a similar extent, the siRNAs that targeted exclusively gRNA significantly stronger suppressed replicating virus (Figure 3D). This finding was further confirmed in an experiment using *wildtype* SARS-CoV-2, which showed an improved knockdown of SARS-CoV-2 gRNA (Figure 3E) leading to enhanced suppression of sgRNAs by ORF1-specific siRNAs (Supplementary Figure S3D). The enhanced viral suppression led to improvements of the metabolic rate of infected cells (Supplementary Figure S3E) and reduced cell death (Figure 3F,G; Supplementary Figure S3F).

In summary, our data showed a concurrent suppression of genomic and subgenomic viral RNAs by siRNAs that targeted the common regions of viral transcripts. On the other hand, the ORF1-specific siRNAs, which solely targeted SARS-CoV-2 gRNA, subdued viral replication and spread more efficiently as compared to siRNAs that additionally targeted sgRNAs.

### Subgenomic RNAs out-compete siRNAs that target the common regions of transcripts leading to a reduced antiviral efficacy

We hypothesized, that the reason for the decreased efficacy of siRNAs that targeted gRNA and sgRNAs could be that they were outnumbered by the highly abundant sgRNAs. In a first approximation, we asked how the level of viral replication would affect knockdown efficacy of siRNAs. We transfected a relatively low concentration (1 nM) of an siRNA that either targeted both, gRNA and sgRNA (N2) or exclusively gRNA (O2) into VeroE6 cells and infected cells with increasing amounts of rSARS-CoV-2-GFP. Interestingly, we found that both siRNAs reduced viral replication to the same extent when cells were infected with a relatively low amount of virus (MOI 0.03). With increasing viral inoculum, however, the sgRNA-targeting siRNA more prominently lost antiviral efficacy than the exclusively gRNA-targeting siRNA (Figure 4A). This was a first indication that out-competition of siRNAs by sgRNAs could indeed be responsible for the reduced antiviral efficacy of siRNAs that target the common regions of transcripts. To substantiate this finding, we asked if increasing siRNA dosages could compensate for this effect. Cells were thus transfected with increasing siRNA concentrations of the same siRNAs and infected with rSARS-CoV-2-GFP. In line with the previous experiment, both siRNAs inhibited viral replication to similar extent when transfected at very high concentrations of 100 nM. With decreasing concentrations, likewise, the sgRNA-targeting siRNA showed a substantial loss of antiviral efficacy which was significantly less distinct for the siRNA that targeted only gRNA (Figure 4B). This added further evidence that competition with sgRNAs impaired antiviral efficacy of siRNAs that target the common regions of transcripts.

The above observations prompted us to acquire a more thorough picture of how SARS-CoV-2 replication impacts the antiviral efficacy of siRNAs at different concentrations. We thus determined the IC_50_ for each siRNA using both, luciferase reporters (Supplementary Figure S4), as well as the SARS-CoV-2 infection model (Supplementary Figure S5). If indeed common region siRNAs would be out-competed by sgRNAs, we would expect higher IC_50_ compared to siRNAs that solely target gRNA. This phenomenon, however, should only appear in the SARS-CoV-2 model as the luciferase reporters do not express sgRNAs. In general, IC_50_ determined using luciferase reporters were considerably lower than those determined in the infection model, most likely as due to the co-transfection of siRNA and reporter plasmid, the majority of cells that expressed luciferase had also received an siRNA. However, this was probably not the case in the infection model, where SARS-CoV-2-infected and siRNA-transfected cells did not necessarily overlap to such a large extent. On the same lines of similar relative knockdown extents (Figure 3D, Supplementary Figure S3B), we also found comparable IC_50_ values for ORF1- or common region siRNAs when tested against luciferase reporters (left graph of Figure 4C). In the infection model, however, common region siRNAs presented significantly higher IC_50_ than ORF1-specific siRNAs (right graph of Figure 4C), as higher siRNA concentrations were necessary to suppress viral replication. In summary, our data show that SARS-CoV-2 replication negatively affected the efficacy of siRNAs which targeted sgRNAs, but not the ones which exclusively targeted gRNA.

To finally prove that the silencing capacity of common region siRNAs was indeed impaired by SARS-CoV-2 replication, we designed a competition experiment. We co-transfected siRNAs targeting the different SARS-CoV-2 regions together with their respective luciferase reporters. After 6h, we infected cells with *wildtype* SARS-CoV-2 and analyzed how SARS-CoV-2 replication would affect silencing of the luciferase reporter (Figure 4D). Of note, in this experimental setting, both SARS-CoV-2 RNAs and mRNA transcribed from a luciferase reporter plasmid can be targeted by the respective siRNAs. Indeed, we found that silencing of luciferase reporters by siRNAs which targeted the common region of transcripts was significantly impaired by SARS-CoV-2 replication. This was not observed for the ORF1-specific siRNA which suppressed the luciferase reporter with same efficacy in both, infected as well as non-infected cells (Figure 4E). We furthermore examined possible confounding factors, such as the siRNA design (Supplementary Table S3 and Figure S6A,B), or the secondary structure of the target region (Supplementary Figure S6C–E), none of which explained the better antiviral activities of siRNAs that targeted solely gRNA.

In summary, our data proves that an impaired RNAi silencing affects siRNAs that targeted sgRNAs leading to a reduced antiviral efficacy.

### *Ex vivo* human lung model confirms the antiviral activity of an ORF1-targeting siRNA therapy

An important factor to consider especially while devising a siRNA-based therapy against RNA viruses is the conservation of the target sites, to enable a broad applicability and minimize the risk of resistance mutations occurring. When analyzing publicly available SARS-CoV-2 sequencing results, we found that the conservation of target sites of our siRNAs varied largely. Interestingly, ORF1-targeting siRNAs showed a significantly higher conservation than siRNAs against the common regions of transcripts (Supplementary Figure S7). The target sites of all three analyzed ORF1-specific siRNAs were conserved to at least 99.55% within all currently circulating strains and within >99.30% of each of the VoC and VoI. The best-performing siRNA, O3, even presented an overall conservation of 99.90% and at least 99.83% within all VoC and VoI (Table 3).

**Table 3.** Conservation of siRNA target sites in circulating SARS-CoV-2 strains. Full-length, high-quality SARS-CoV-2 sequencing results were retrieved from the GISAID EpiCoV™ Database (www.gisaid.org) and analyzed for the presence of the siRNA target sites. To estimate conservation within all currently circulating strains (‘Any variant’), the 100,000 latest submissions until October 26^th^ 2021 were included without restricting to a specific variant. Lineages defined by WHO as Variants of Concern (VoC) or Variants of Interest (VoI) were separately downloaded and analyzed accordingly. For VoC, only the latest 20,000 submissions, and for VoI, all available sequences were considered. VoC and VoI are labeled according to WHO nomenclature, for details regarding the included lineages according to the Pango nomenclature system see materials & methods. n, number of analyzed full-length SARS-CoV-2 sequences

		Variants of concern				Variants of interest	
Variant	Any variant	Alpha	Beta	Gamma	Delta	Lambda	Mu
Sequences (n)	100 000	20 000	20 000	20 000	20 000	848	5889
**siRNA**	**Conservation of target site** (Only perfect matches in %)						
L1	94.92	95.10	91.15	96.65	93.43	98.23	92.96
L2	39.53	37.34	36.16	29.81	47.39	22.40	28.76
L3	24.11	23.32	26.06	17.43	15.95	16.98	13.04
O1	99.55	99.33	99.83	99.42	99.51	99.76	99.59
O2	99.65	99.46	99.45	99.66	99.33	99.88	99.54
O3	99.90	99.95	99.88	99.93	99.91	100	99.83
N1	99.54	99.60	99.71	99.71	99.55	99.64	99.88
N2	99.68	99.79	99.90	99.89	99.50	99.52	99.06
N3	99.50	99.69	99.77	98.70	99.67	99.29	99.52
U1	96.49	96.89	97.20	91.16	93.78	92.57	90.25
U2	86.64	84.74	86.05	59.20	82.78	89.62	81.28
U3	24.53	38.39	33.49	25.95	35.98	38.44	28.83

**Figure 5.**
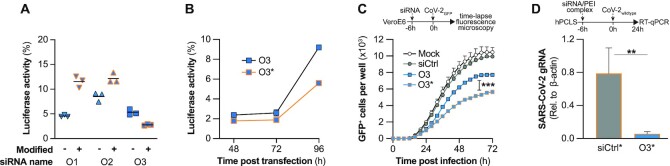
Chemically modified siRNA inhibits SARS-CoV-2 replication *ex vivo* in the human lung. (**A**) ORF1-targeting siRNAs were chemically modified using a clinically validated chemistry (for details, see Material and Methods and Supplementary Table S4) and the activity compared to chemically non-modified versions of the siRNAs using luciferase reporters. For this, siRNAs and luciferase reporter plasmids were co-transfected into HEK293T cells and after 24 h luciferase activities determined. Values were normalized to a control group transfected with the respective luciferase reporter and the control siRNA with identical chemistry. (**B**) Effect of chemical modifications on the duration of RNAi-silencing by siRNA O3 was compared using the same experimental setup as in (A), and luciferase activity was determined at indicated time points. (**C**) Antiviral activity of the modified and non-modified version of siRNA O3 were compared using the rSARS-CoV-2-GFP model. siRNAs were transfected into VeroE6 at a concentration of 50nM. 6h later, cells were infected with rSARS-CoV-2-GFP (MOI1), and GFP^+^ cells were quantified using the Incucyte S3 system. (**D**) To validate the approach in a highly relevant model of the human lung, the chemically modified siRNA O3 was complexed with polyethylenimine (PEI), and transfected into human precision cut lung slices (hPCLS; 100nM), which were infected with *wildtype* SARS-CoV-2 (MOI 1) 6h later. RNA was extracted from hPCLS harvested 24h p.i. and viral replication quantified by RT-qPCR for SARS-CoV-2 gRNA (normalized to β-actin expression). Experiments shown in (A–C) were performed using three biological replicates, (D) using five replicates. Horizontal bars in (A, D) indicate mean, error bars in (B–D) S.E.M. n.s., non-significant; **P < 0.01; ***P < 0.001.

As non-modified siRNAs are prone to nuclease digestion, we tested chemically stabilized versions of our ORF1-targeting siRNAs using a modification pattern (46), that is also employed by the recently approved Lumasiran (47). While the silencing activity of O1 and O2 were negatively affected by the introduction of these chemical modifications, the modified version of O3 (O3*) presented even an enhanced activity against the luciferase reporter (Figure 5A). In combination with the finding that O3 targeted also the most conserved viral target of all analyzed siRNAs, it prompted us to select O3* for further experiments. In line with the expected increased stability, the modified version of O3 revealed an even stronger pronounced benefit at later time points (Figure 5B). Consequently, O3* also inhibited SARS-CoV-2 replication significantly stronger than the non-modified siRNA (Figure 5C).

To test the relevance of our findings, we aimed to validate O3* in a more realistic model of the human lung and opted for human precision cut lung slices (hPCLS). PCLS are a complex *ex vivo* 3D tissue culture model of primary human lung cells and thus constitute a highly physiological model to evaluate siRNA delivery to the human lung and to study human respiratory viruses (58). Also, we delivered our siRNAs using PEI this time, which has a well characterized toxicity profile allowing *in vivo* application (56) and can better be nebulized than liposomal formulations (59), an important characteristic for a lung-directed therapy. hPCLS were infected with wildtype SARS-CoV-2 6h after siRNA application, and effects on viral replication were assessed after 24 h by RT-qPCR. Indeed, also in this highly realistic model of the human lung, siRNA O3* significantly inhibited SARS-CoV-2 replication by 92.8% compared to the control siRNA-treated group (Figure 5D).

In summary, we show that factors which might proof crucial for clinical translation can be applied to ORF1-targeting siRNAs, including a high conservation of the target site, the stabilization via chemical modifications, as well as a formulation which supports application by inhalation. The resulting therapy strongly inhibited SARS-CoV-2 replication *ex vivo* in explants of the human lung, underlining the relevance of our findings.

## DISCUSSION

A promising approach to develop antiviral therapies against SARS-CoV-2 constitute siRNAs, which is pursued by several academic and industry groups. First proof-of-concept studies presented that SARS-CoV-2 can be targeted with siRNAs. Until today, however, there is no in-depth investigation which coronaviral replication steps can be targeted with siRNAs, which is not even available for other positive sense RNA viruses. By systematically analyzing the individual replication steps following cell entry, we found that siRNAs, when given in a prophylactic setting, can target the genome of SARS-CoV-2 at an early replication step and halt replication before start of transcription, preventing virus-induced cell death. To our surprise, targeting solely gRNA resulted in a stronger antiviral efficacy than a simultaneous targeting of gRNA and sgRNA. We show that the impaired RNAi silencing affecting siRNAs that target gRNA and sgRNAs results from an out-competition by the highly abundant sgRNAs. This notion appears especially plausible as Kim *et al.* showed that roughly 2/3rd of the transcriptome of infected cells are made up of SARS-CoV-2 RNAs of which almost all contain the targeted sequences (38).

Our findings on a first look might contradict a previous report which described that targeting the leader sequence of SARS-CoV-1 with siRNAs would be more efficacious than targeting the S ORF (12). Several factors could explain differences found in our study. First of all, SARS-CoV-1, which—while being the closest related virus—has an amino acid sequence homology of only between 40 and 94% depending on the ORF (60), thus findings might not be applicable to SARS-CoV-2. Also, Li *et al.* compared an siRNA targeting the Leader sequence to siRNAs targeting the S gene, which does not only exist in gRNA, but also in at least a fraction of sgRNAs. Third, Li *et al.* compared only a single Leader-specific siRNA to two S-specific siRNAs questioning if the finding can be generalized to the target region or if intra-individual differences of siRNA activity were responsible for the observed differences.

Along these lines, the question arises if differences between siRNA activities, in contrast to general differences between target regions, could also explain why ORF1-siRNAs were most efficient in our study. Given that ORF1 constitutes roughly 2/3rd of the viral genome, the bigger genomic space compared to the common regions might have allowed to select more efficient siRNAs. This explanation, however, appears unlikely as during the design and preselection of the siRNAs, we prioritized to acquire siRNAs with similar activity for each target region over selecting the most efficient siRNAs. As a result, we chose siRNAs for which a similar efficacy was predicted and which additionally showed comparable knockdown of luciferase reporters. While ORF1-specific siRNAs suppressed luciferase reporters to a similar degree as the common region siRNAs, they were superior in inhibiting replicating virus. This proves that a virological factor, rather than more efficient siRNA sequences, was responsible for the better antiviral efficacy of ORF1-specific siRNAs. Nonetheless, activities of different siRNA sequences can differ strongly. Thus, we cannot exclude that an siRNA against any SARS-CoV-2 genomic region might proof highly efficacious in inhibiting viral replication. Also, as we did not investigate any other target region beside the leader sequence, ORF1, N, or the 3′UTR, we cannot exclude that targeting another genomic region of SARS-CoV-2 could prove to be superior to targeting ORF1.

Another factor which might influence the antiviral activity of siRNAs could be the accessibility of the viral RNA. One the one side, certain replication steps might occur within cellular compartments, which we believe is the most plausible explanation why negative sense RNA was not accessible for siRNA silencing. As negative sense RNAs do not encode for proteins, there is no need to export them to the cytosol. However, as probably no RISC complexes are present within the ROs, activity of siRNA is restricted to viral RNAs which have either not yet entered ROs (gRNA of incoming virus), or positive sense sgRNAs that are exported from ROs for translation. Another factor which can influence the accessibility of RNAs are secondary structures (61), which are especially important characteristics of viral RNAs. While we found no correlation of predicted secondary structures with the higher antiviral activity of ORF1-targeting siRNAs, further factors, such as the coverage of specific genomic regions by viral or host accessory proteins, could potentially also have an impact.

It furthermore needs to be mentioned, that our study did not employ *in vivo* experiments. While we used two different cell culture infection models with varying conditions such as time points of siRNA application, siRNAs dosages or viral inoculum sizes, we still cannot exclude, that factors play out crucial during *in vivo* application which could not be addressed in these models. Nonetheless, by verifying the antiviral activity of our lead siRNA in hPCLS, we validated our approach in a model system which in certain aspects can be considered as even more relevant for translational aspects than the available animal models. As hPCLS are explants of human lungs, they contain all cell types (including resident immune cells) and the physiologic structural architecture that is characteristic for the human lung. This comprises several factors with potential influence on efficacy of a siRNA therapy, such as the cell polarization, mucus production, or the innate immune system.

For clinical translation of siRNA-based therapies, several additional factors need to be considered. One issue is the possible occurrence of escape mutations that render the virus resistant to therapy. Here, it is assumed that a high conservation of the specific genomic region goes along with an essential function for the virus, limiting the likelihood of such mutations occurring. While during the design of our siRNAs, we originally did not take the conservation of target sites into account, the targets of our ORF1-specific siRNAs were significantly more conserved than siRNAs that targeted sgRNAs, which is supported by the fact that ORF1 shows a relatively high conservation compared to other regions of the SARS-CoV-2 genome (62). The target site of our most efficient siRNA, O3, even showed a conservation of 99.9% in currently circulating SARS-CoV-2 strains. These findings strongly support the assumption that an ORF1-specific siRNA drug candidate with a broad applicability and a high resistance barrier could be developed.

Another important factor is the resistance of the siRNA to nucleases, which are found throughout different body compartments and can minimize siRNA activity especially *in vivo*. Using a clinically validated chemical modification pattern, we show that our most promising siRNA candidate O3 gained a higher and more durable RNAi activity by introducing these modifications, leading to an enhanced antiviral efficacy. Moreover, the chemically modified siRNA could successfully be complexed with PEI, which most likely constitutes a further protection from nucleases.

Clinical application of siRNA-based approaches furthermore crucially depends on siRNA delivery (63–66). Especially the identification of the optimal carrier and administration route is an important factor. While pulmonary delivery can be achieved by intranasal (i.n.) or inhalation administration, i.n. administration was chosen as the delivery route in Alnylam's early attempts of delivering siRNA against RSV (67). The big advantage of i.n. delivery is the possibility of administering a liquid formulation as nose drops without the need of nebulizing the formulation. This is particularly of impact for liposomal formulations as they do not withstand shear forces and temperature-related stress during nebulization (59). The biggest disadvantage of i.n. administration, however, is the low pulmonary bioavailability of the administered dose, while a large proportion is swallowed and degraded (68). Inhalation delivery, in contrast, requires aerosol development of a mist or dry powder. For nebulization of macromolecules such as siRNA, vibrating mesh nebulizers are preferred for decreased effects on biomolecule stability (64). Dry powder inhalation offers the advantages of long shelf-lives and enhanced stability of nucleic acid formulations against chemical, physical and microbial factors (65) but faces engineering challenges when nucleic acids nanoformulations need to be transferred into dry powders (69). Such nanoformulations are, however, particularly important for pulmonary delivery where free nucleic acids do not efficiently diffuse through the mucus barrier for subsequent uptake into the epithelium (70). Numerous siRNA nanoformulations exist based on polymers, lipids, peptides and inorganic materials (71), each of which can be improved in efficiency and specificity with different surface modifications such as targeting ligands or membrane-active substances (66). PEI polymer has widely been investigated as delivery system for siRNA (56). Thanks to its positive surface charge it can be used to complex negatively charged nucleic acids. PEI ensures high encapsulation efficiency of siRNA even at low N/P ratio, it protects the cargo against degradation by RNases and confers higher transfection efficiency in comparison to free siRNA (72). In this study, siRNA O3*/PEI polyplexes confirmed the activity of the siRNA against SARS-CoV-2 in a relevant *ex vivo* model, the hPCLS, which closely mimic the anatomy of the respiratory tract.

To our knowledge, there is so far no equally detailed analysis of RNAi-targetable replication steps and RNA species for any positive sense RNA virus. Thus, our results might also be of relevance beyond SARS-CoV-2. The reduction of cytopathic effects achieved by antiviral siRNAs could be crucial, as endothelial injury has been proposed to trigger pathology in lethal COVID-19 cases (4). Along this line, early therapy starts, or possibly even prophylactic application of siRNAs appears as major benefit. Exclusive targeting of gRNA was advantageous over targeting sgRNAs additionally which could be a valuable information for designing siRNAs and treatment regimens in clinical studies. Taken together, our study confirms that siRNA-based strategies could allow to develop potent antivirals to reduce pathology of COVID-19, encouraging academia and industry to proceed with ongoing efforts.

## DATA AVAILABILITY

All data supporting the findings of this study are available within the article and the supplementary information files. The SARS-CoV-2 reference sequence used as a template for siRNA design is available at the NCBI RefSeq database under the accession number: NC_045512.2. The sequence of the SARS-CoV-2 variant used for infection experiments with *wildtype* virus is available on the GISAID database (https://www.gisaid.org/) under the accession ID: EPI_ISL_582134.

## Supplementary Material

gkab1248_Supplemental_File

## Notes

Present address: Suliman Qadir Afridi, Fraunhofer Institute for Cell Therapy and Immunology, Perlickstraße 1, 04103 Leipzig, Germany.
