# Supplementary material for: Targeting genomic SARS-CoV-2 RNA with siRNAs allows efficient inhibition of viral replication and spread
Source: Nucleic Acids Res. 2021 Dec 20;50(1):333–49. doi: 10.1093/nar/gkab1248 (PMC8754636; doi:10.1093/nar/gkab1248)
Supplement: gkab1248_Supplemental_File [file gkab1248_supplemental_file.pdf]

Supplementary information for manuscript:

Targeting genomic SARS-CoV-2 RNA with siRNAs allows efficient inhibition of viral replication and spread

Name	Sense strand (5'-3')	Antisense strand (5'-3')
N12	GCCAAAAGGCUUCUACGuATT	UGCGUAGAAGCCUUUUGGCAA
N13	CCUCAUCACGUAGUCGuAATT	UUGCGACUACGUGAUGAGGAA
N14	GUAACACAAGCUUUCGGuATT	UGCCGAAAGCUUGUGUUACAU
N15	CGAAAUGCACCCCGCAUUATT	UAAUGCGGGGUGCAUUUCGCU
N16	CGAAGAGCUACCAGACGAATT	UUCGUCUGGUAGCUCUUCGGU
N17	UCCUCAUCACGUAGUCGCAACTT	GUUGCGACUACGUGAUGAGGATT
N18	AUCACAUUGGCACCCGCAAUCTT	GAUUGCGGGUGCCAAUGUGAUTT
N19	UGGUGCUAACAAAGACGGCAUTT	AUGCCGUCUUUGUUAGCACCATT
N20	AAGCCUCGGCAAAAACGUACUTT	AGUACGUUUUUGCCGAGGCUUTT
N21	GCCUGGGGUUUUAGUCGuUTT	AGCGACUAAAACCCCAGGCAA
N22	CUGAAGGGAUACCACGAUUTT	AAUCGUGGUAUCCCUUCAGGU
N23	GUCUACUGGUUUAACCGAUTT	AUCGGUUAAACCAGUAGACCU
N24	GUUUUCCGAAGAUGCGUuUTT	AGACGCAUCUUCGGAAAACCG

Supplementary Table S1. Sequences of additional siRNAs that were screened regarding their activity against the negative- or positive-sense Nucleocapsid gene. siRNA duplexes were designed with occasional G:U wobbles at the 5' end of the antisense strand, as indicated by small lettered 'u'. N12-24, N-specific siRNAs 12-24; A = Adenine; C = Cytosine; G = Guanine; U = Uracil; T = Thymine

Name	Forward strand (5'-3')	Reverse strand (5'-3')
Negative-<br>sense<br>Nucleocapsid<br>gene	TCGAGTACAGACTATTGCCTGGGGTTTTAGTCGCT<br>TTGCGTGGGGCGTAATGCAAACCACCTGGGAGTC<br>TAAGTTGACCGTCATTGGTCTTGCCTCTTGCGTCA<br>CCCCGCGCTAGTTTTGTTGCAGCCGGGGTTCCAA<br>ATGGGTTATTATGACGCAGAACCAAGTGGCGAGA<br>GTGAGTTGTACCGTTCCTTCTGGAATTTAAGGGAG<br>CTCCTGTTCCGCAAGGTTAATTGTGGTTATCGTCA<br>GGTCTACTGGTTTAACCGATGATGGCTTCTCGATG<br>GTCTGCTTAAGCACCACCACTGCCCTTTTACTTTC<br>TAGAGTCAGGTTCTACCATAAAGATGATGGATCCT<br>TGACCCGGTCTTCGACCTGAAGGGATACCACGAT<br>TGTTTCTGCCGTAGTATACCCAACGTTGACTCCCT<br>CGGAACTTATGTGGTTTTCTAGTGTAACCGTGGGC<br>GTTAGGACGATTGTTACGACGTTAGCACGATGTTG<br>AAGGAGTTCCTTGTTGTAACGGTTTTCCGAAGATG<br>CGTCTTCCCTCGTCTCCGCCGTCAGTTCGGAGAA<br>GAGCAAGGAGTAGTGCATCAGCGTTGTCAAGTTC<br>TTTAAGTTGAGGTCCGTCGTCATCCCCTTGAAGAG<br>GACGATCTTACCGACCGTTACCGCCACTACGACG<br>AGAACGAAACGACGACGAACTGTCTGAGTTGGTC<br>GAACTCTCGTTTTACAGACCATTTCCGGTTGTTGT<br>TGTTCCGGTTTGACAGTGATTCTTTAGACGACGAC<br>TCCGAAGATTCTTCGGAGCCGTTTTTGCATGACGG<br>TGATTTCGTATGTTACATTGTGTTCGAAAGCCGTC<br>TGCACCAGGTCTTGTTTGGGTTCCTTTAAAACCCC<br>TGGTCCTTGATTAGTCTGTTCCTTGACTAATGTTTG<br>TAACCGGCGTTTAACGTGTTAAACGGGGGAGGCG<br>ATCGCGCAAGAAGCCTTACAGCGCGTAACCGTAC<br>CTTCAGTGTGGAAGCCCTTGCACCAACTGGATGT<br>GTCCCCGGTAGTTTAACCTACTGTTTCTAGGTTTA<br>AAGTTTCTAGTTCAGTAAAACGACTTATTCGTATAA<br>CTGCGTATGTTTTGTAAGGGTGGTTGTCTCGGATT<br>TTTCCTGTTTTTCTTCTTCCGACTACTTTGAGTTCG<br>GAATGGCGTCTCTGTCTTCTTTGTCGTTTGACACT<br>GAGAAGAAGGACGACGGCTAAACCTACTAAAGAG<br>GTTTGTTAACGTTGTTAGGTACTCGTCACGACTGA<br>GTTGAGTCCGGGC	GCCCGGACTCAACTCAGTCGTGACGAGTACCTAACA<br>ACGTTAACAAACCTCTTTAGTAGGTTTAGCCGTCGT<br>CCTTCTTCTCAGTGTCAAACGACAAAGAAGACAGAG<br>ACGCCATTCCGAACTCAAAGTAGTCGGAAGAAGAAA<br>AACAGGAAAAATCCGAGACAACCACCCTTACAAAAC<br>ATACGCAGTTATACGAATAAGTCGTTTTACTGAACTA<br>GAAACTTTAAACCTAGAAACAGTAGGTTAAACTACC<br>GGGGACACATCCAGTTGGTGCAAGGGCTTCCACAC<br>TGAAGGTACGGTTACGCGCTGTAAGGCTTCTTGCGC<br>GATCGCCTCCCCCGTTTAACACGTTAAACGCCGGTT<br>ACAAACATTAGTCAAGGAACAGACTAATCAAGGACC<br>AGGGGTTTTAAAGGAACCCAAACAAGACCTGGTGCA<br>GACGGCTTTCGAACACAATGTAACATACGAAATCAC<br>CGTCATGCAAAAACGGCTCCGAAGAATCTTCGGAGT<br>CGTCGTCTAAAGAATCACTGTCAAACCGGAACAACA<br>ACAACCGGAAATGGTCTGTAAAACGAGAGTTCGACC<br>AACTCAGACAGTTCGTCGTCGTTTCGTTCTCGTCGT<br>AGTGGCGGTAACGGTCGGTAAGATCGTCCTCTTCAA<br>GGGGATGACGACGGACCTCAACTTAAAGAACTTGAC<br>AACGCTGATGCACTACTCCTTGCTCTTCTCCGAACT<br>GACGGCGGAGACGAGGGAAGACGCATCTTCGGAAA<br>ACCGTTACAACAAGGAACTCCTTCAACATCGTGCTA<br>ACGTCGTAACAATCGTCCTAACGCCCACGGTTACAC<br>TAGAAAACCACATAAGTTCCGAGGGAGTCAACGTTG<br>GGTATACTACGGCAGAAACAATCGTGGTATCCCTTC<br>AGGTCGAAGACCGGGTCAAGGATCCATCATCTTTAT<br>GGTAGAACCTGACTCTAGAAAGTAAAAGGGCAGTG<br>GTGGTGCTTAAGCAGACCATCGAGAAGCCATCATCG<br>GTTAAACCAGTAGACCTGACGATAACCACAATTAAC<br>CTTGCGGAACAGGAGCTCCCTTAAATTCCAGAAGGA<br>ACGGTACAACTCACTCTCGCCACTTGGTTCTGCGTC<br>ATAATAACCCATTTGGAACCCCGGCTGCAACAAAAC<br>TAGCGCGGGGTGACGCAAGAGGCAAGACCAATGAC<br>GGTCAACTTAGACTCCCAGGTGGTTTGCATTACGCC<br>CCACGCAAAGCGACTAAAACCCCAGGCAATAGTCTG<br>TACTCGA
L1 target	TCGAGTCTGTTCTCTAAACGAACTGC	GGCCGCAGTTCGTTTAGAGAACAGAC
L2 target	TCGAGCCAACCAACTTTCGATCTCGC	GGCCGCGAGATCGAAAGTTGGTTGGC
L3 target	TCGAGAAACCAACCAACTTTCGATGC	GGCCGCATCGAAAGTTGGTTGGTTTC
O1 target	TCGAGAACCAAATGTGCCTTTCAACTGC	GGCCGCAGTTGAAAGGCACATTTGGTTC
O2 target	TCGAGTTGTTACATGCACCATATGGAGC	GGCCGCTCCATATGGTGCATGTAACAAC
O3 target	TCGAGATGGTACTTGGTAGTTTAGCTGC	GGCCGCAGCTAAACTACCAAGTACCATC
N1 target	TCGAGGCCAAAAGGCTTCTACGCAGC	GGCCGCTGCGTAGAAGCCTTTTGGCC
N2 target	TCGAGGAATAAGCATATTGACGCAGC	GGCCGCTGCGTCAATATGCTTATTCC
N3 target	TCGAGCGCTTCAGCGTTCTTCGGAATGC	GGCCGCATTCCGAAGAACGCTGAAGCGC
U1 target	TCGAGATCTTTAATCAGTGTGTAACAGC	GGCCGCTGTTACACACTGATTAAAGATC
U2 target	TCGAGGCCCTAATGTGTAAAATTAATGC	GGCCGCATTAATTTTACACATTAGGGCC
U3 target	TCGAGCCCATGTGATTTTAATAGCTTGC	GGCCGCAAGCTATTAAAATCACATGGGC

Supplementary Table S2. Oligonucleotides used for cloning of luciferase reporters. L1-3; Leader-sequence specific siRNAs 1-3; O1-3, ORF1-specific siRNAs 1-3; N1-3, N-specific siRNAs 1-3; U, 3´UTR-specific siRNAs 1-3; A = Adenine; C = Cytosine; G = Guanine; T = Thymine

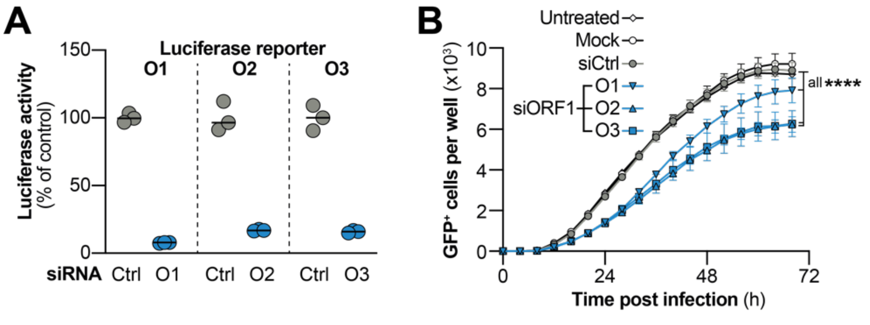

Supplementary Figure S1. Long-term effect of ORF1-specific siRNAs on viral spread and cell viability. (**A**) siRNAs targeting ORF1 (O1-O3) or as control GFP (siCtrl) were co-transfected with luciferase reporters carrying the ORF1 siRNA target sites into HEK293T cells. *Renilla* luciferase activity was measured 48h post transfection and normalized to Firefly luciferase activity. (**B**) Same experiment as shown in Figure 1B demonstrating time kinetic until 72h p.i. VeroE6 cells were transfected with siRNAs targeting ORF1 16h before infection with recombinant, GFP-expressing SARS-CoV-2 (rSARS-CoV-2-GFP; MOI 1) and number of GFP$^+$ positive cells determined by automated quantification using the Incucyte S3 software. (**C**) VeroE6 cells were transfected with siRNAs 6h before infection with *wildtype* SARS-CoV-2 (MOI 0.1) and 24h later cell viability determined by measuring conversion from resazurin to resorufin using a fluorometer. (B) Mean of triplicates for each treatment group +/- SEM is shown. Bars in (A) indicate median of three replicates and in (C) mean of five replicates per treatment group. Statistical differences were calculated using (B) Repeated Measures One-Way Anova or (C) regular One-Way Anova with Dunnett´s multiple comparison correction. O1-3, ORF1-specific siRNAs 1-3; ****, p<0.0001

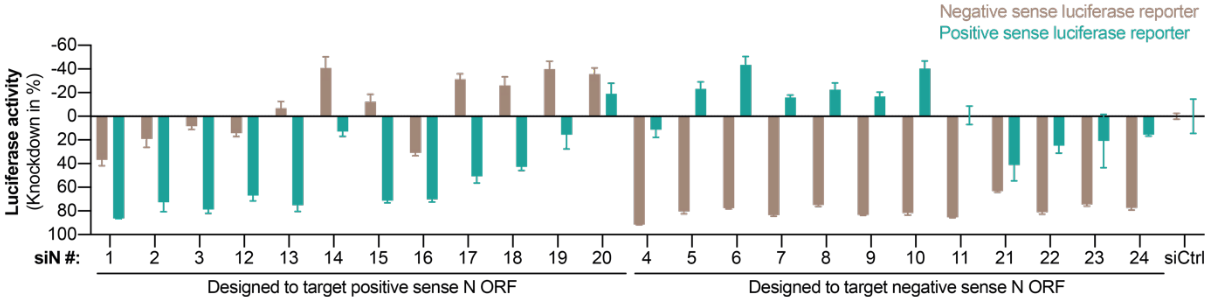

Supplementary Figure S2. Prescreening with luciferase reporters to identify siRNAs with selective activity against positive or negative sense N ORF. HEK293T cells were co-transfected with siRNAs and reporter plasmids expressing Renilla luciferase with either negative or positive sense N ORF cloned into the 3'UTR. 48h after transfection, cells were lysed and luciferase activity measured. Graph shows reduction of luciferase activity by the SARS-CoV-2 specific siRNA compared to the same reporter co-transfected with the control siRNA. siRNAs N1-11 were selected for further studies. Bars indicate mean of triplicates and error bars SEM.

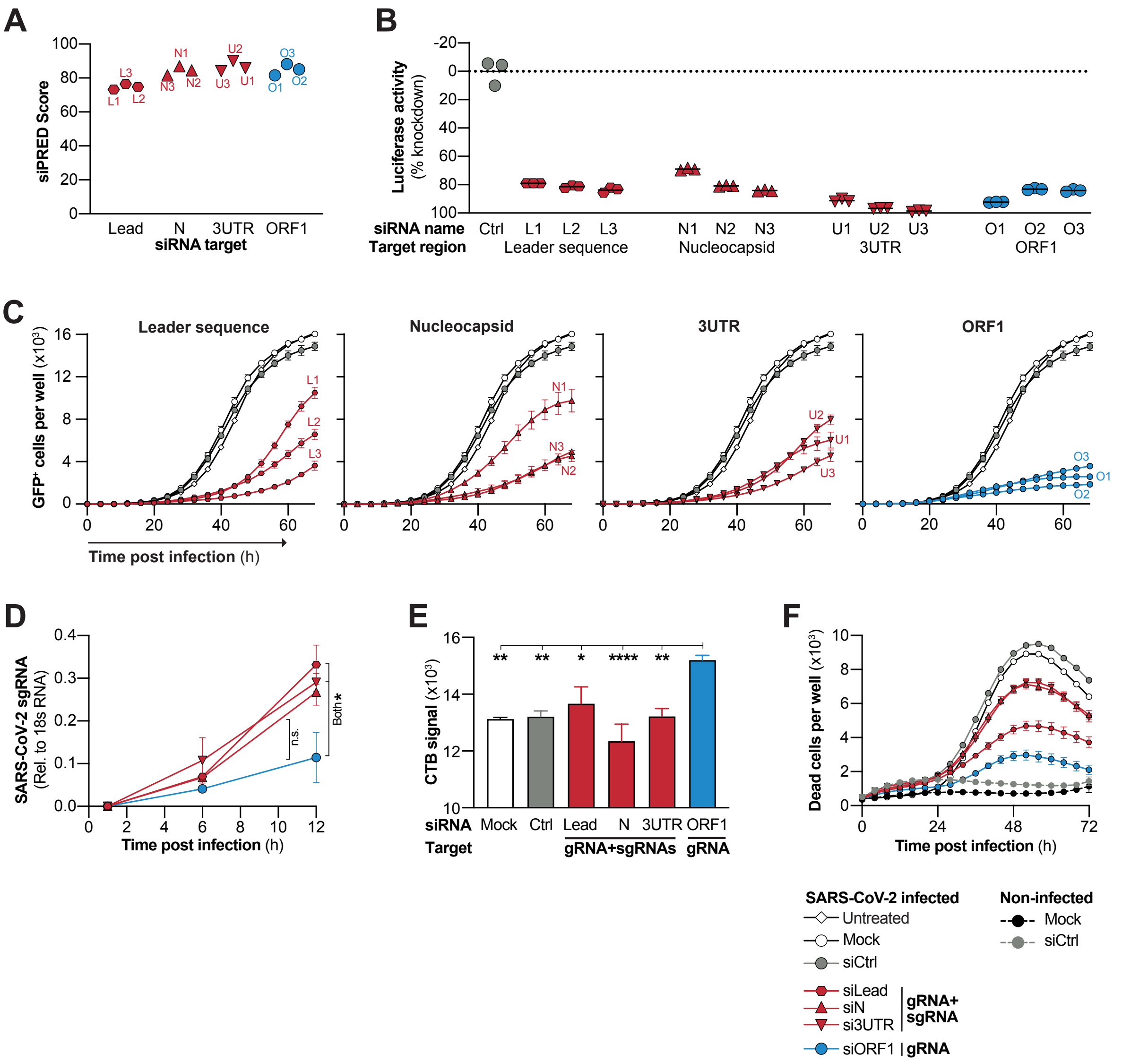

Supplementary Figure S3. siRNAs that target exclusively SARS-CoV-2 gRNA have a more profound effect on viral replication and cytopathy than siRNAs that additionally target sgRNAs. (**A**) *In-silico* prediction of siRNA activity using siPRED online tool (http://predictor.nchu.edu.tw/siPRED/index.php; maximum = 100). (**B**) HEK293T cells were co-transfected with siRNAs and the respective luciferase reporter and luciferase activity measured after 48h. Graph shows values normalized to the same luciferase reporter treated with a control siRNA specific for GFP. (**C**) VeroE6 cells were infected with rSARS-CoV-2-GFP (MOI 1) 3h before transfecting siRNAs against indicated target regions. GFP⁺ cells were quantified every 4h using the Incucyte S3 analysis software. (**D-F**) VeroE6 cells were transfected with siRNA pools against indicated target regions, and 6h later infected with *wildtype* SARS-CoV-2 (MOI 0.1). (**D**) SARS-CoV-2 sgRNA was quantified from cell lysates at indicated time points using RT-qPCR. (**E**) Cell viability was determined after 24h by measuring conversion from resazurin to resorufin using a fluorometer. (**F**) Time kinetic of dead cells over a period of 3 days. Dead cells were quantified using the Incucyte® Cytotox Red Dye and the Incucyte S3. Statistical differences were calculated using (D) repeated measures Anova or (E) One-Way Anova with Dunnett´s multiple comparison correction. Mean and SEM of three biological replicates are shown in (C, D, F) and of five biological replicates in (E). L1-3; Leader-sequence specific siRNAs 1-3; N1-3, N-specific siRNAs 1-3; U, 3´UTR-specific siRNAs 1-3; O1-3, ORF1-specific siRNAs 1-3; n.s., non significant; *, p<0.05; **, p<0.01; ***, p<0.001; ****, p<0.0001

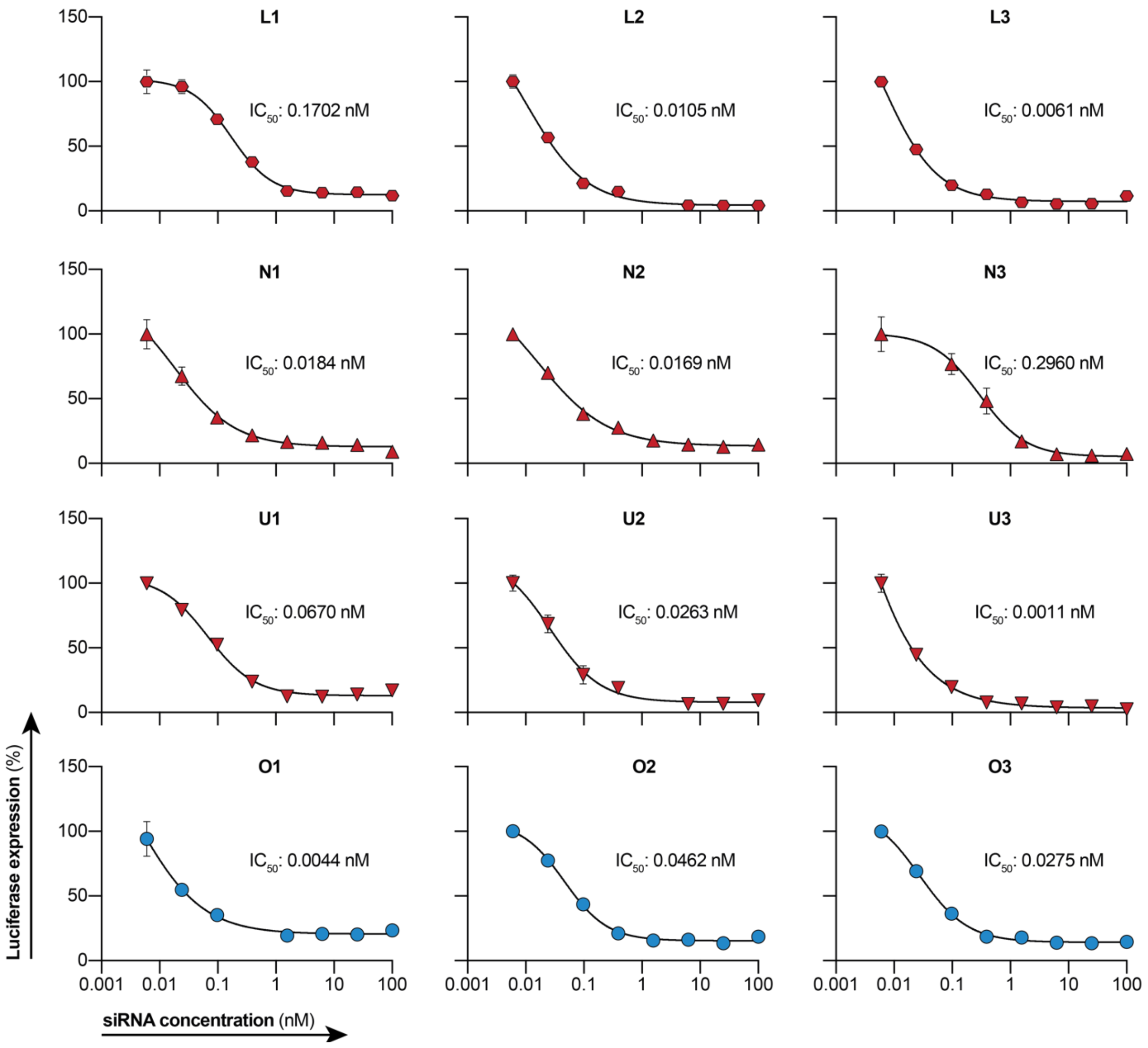

Supplementary Figure S4. Determination of mean inhibitory concentrations (IC_{50}) of siRNAs using luciferase reporters. HEK293T cells were co-transfected with 200 ng of psiCHECK2 luciferase reporters and decreasing concentrations of siRNAs (100, 25, 6.25, 1.56, 0.39, 0.098, 0.024 and 0.006 nM) and relative *Renilla* luciferase activity determined after 48h. IC_{50} values were calculated using non-linear regression (curve fit) in GraphPad 9.0. Mean of three biological replicates ±SEM is shown; L1-3; Leader-sequence specific siRNAs 1-3; N1-3, N-specific siRNAs 1-3; U, 3'UTR-specific siRNAs 1-3; O1-3, ORF1-specific siRNAs 1-3

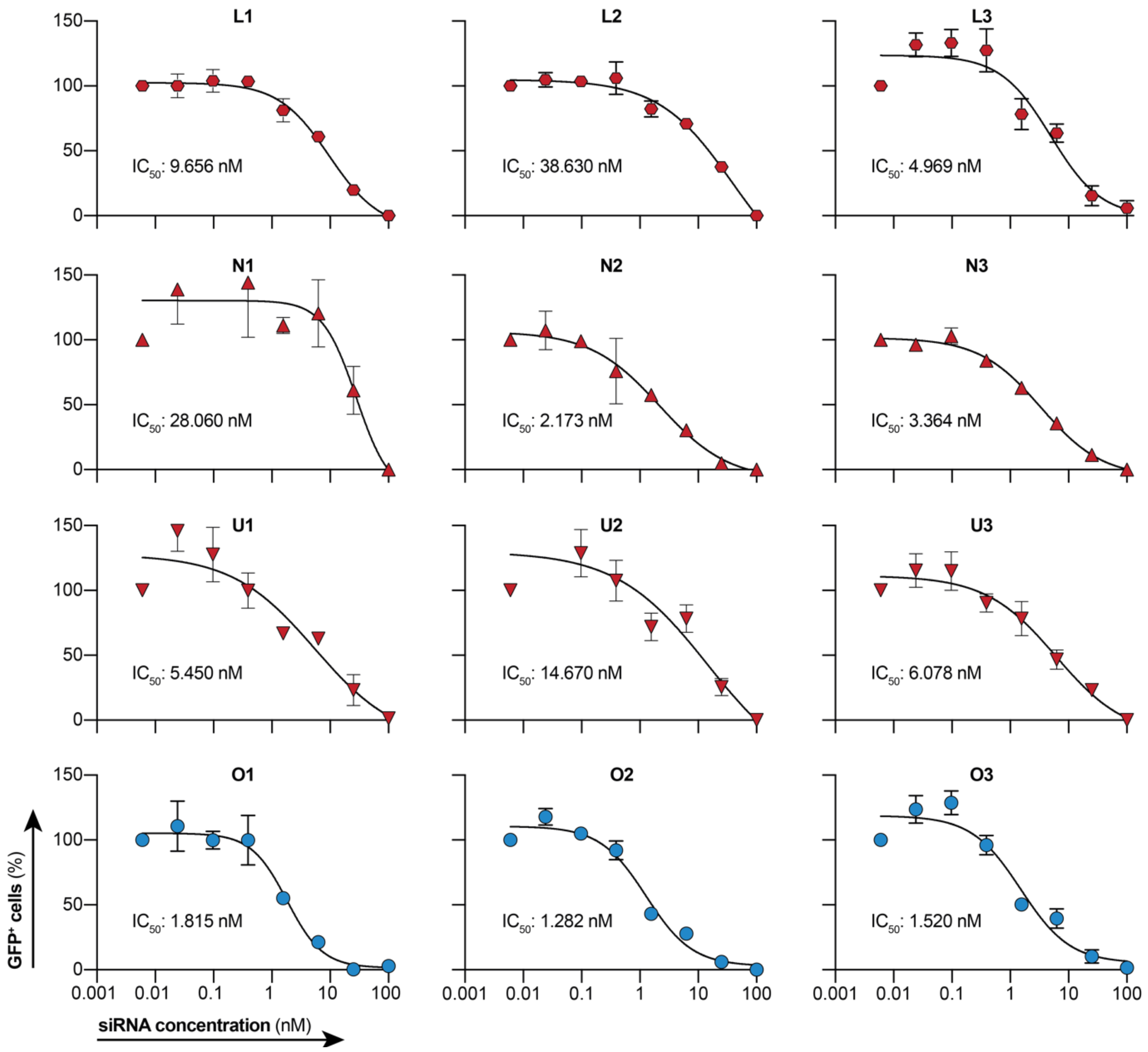

Supplementary Figure S5. Determination of mean inhibitory concentrations (IC_{50}) of siRNAs using the rSARS-CoV-2-GFP infection model. VeroE6 cells were transfected with decreasing concentrations of siRNAs (100, 25, 6.25, 1.56, 0.39, 0.098, 0.024 and 0.006 nM) and infected with rSARS-CoV-2 (MOI 1) after 6h. GFP^+ cells were determined by the Incucyte software 24h p.i. and IC_{50} values calculated using non-linear regression (curve fit) in GraphPad 9.0; Mean of three biological replicates ±SEM is shown; L1-3; Leader-sequence specific siRNAs 1-3; N1-3, N-specific siRNAs 1-3; U, 3'UTR-specific siRNAs 1-3; O1-3, ORF1-specific siRNAs 1-3

Name	Sense strand (5'-3')	Antisense strand (5'-3')	Length (nt)	
			Sense	Antisense
L1	UCUGUUCUCUAAACGAACUTT	AGUUCGUUUAGAGAACAGAUC	21	21
L2	CCAACCAACUUUCGAUCUCTT	GAGAUCGAAAGUUGGUUGGUU	21	21
L3	AAACCAACCAACUUUCGAUTT	AUCGAAAGUUGGUUGGUUUGU	21	21
O1	CCAAAUGUGCCUUUCAACUTT	AGUUGAAAGGCACAUUUGGUU	21	21
O2	GUUACAUGCACCAUAUGGATT	UCCAUAUGGUGCAUGUAACAA	21	21
O3	GGUACUUGGUAGUUUAGCUTT	AGCUAAACUACCAAGUACCAU	21	21
N1	GAAUAAGCAUAUUGACGCATT	UGCGUCAAUAUGCUUAUUCAG	21	21
N2	CAAAUUGGCUACUACCGAATT	UUCGGUAGUAGCCAAUUUGGU	21	21
N3	CUUCAGCGUUCUUCGGAAUTT	AUUCCGAAGAACGCUGAAGCG	21	21
U1	CUUUAAUCAGUGUGUAACATT	UGUUACACACUGAUUAAAGAU	21	21
U2	CCUAAUGUGUAAAAUUAAUTT	AUUAAUUUUACACAUUAGGGC	21	21
U3	CAUGUGAUUUUAAUAGCUUTT	AAGCUAUUAAAAUCACAUGGG	21	21
siLuc	CGUACGCGGAAUACUUCGATT	UCGAAGUAUUCCGCGUACGUG	21	21

Supplementary Table S3. Sequences of siRNAs used in the study with identical design, including same length and not containing wobbles. nt, nucleotides; L1-3; Leader-sequence specific siRNAs 1-3; O1-3, ORF1-specific siRNAs 1-3; N1-3, N-specific siRNAs 1-3; U, 3'UTR-specific siRNAs 1-3; Luc = Firefly Luciferase; A = Adenine; C = Cytosine; G = Guanine; U = Uracil; T = Thymine

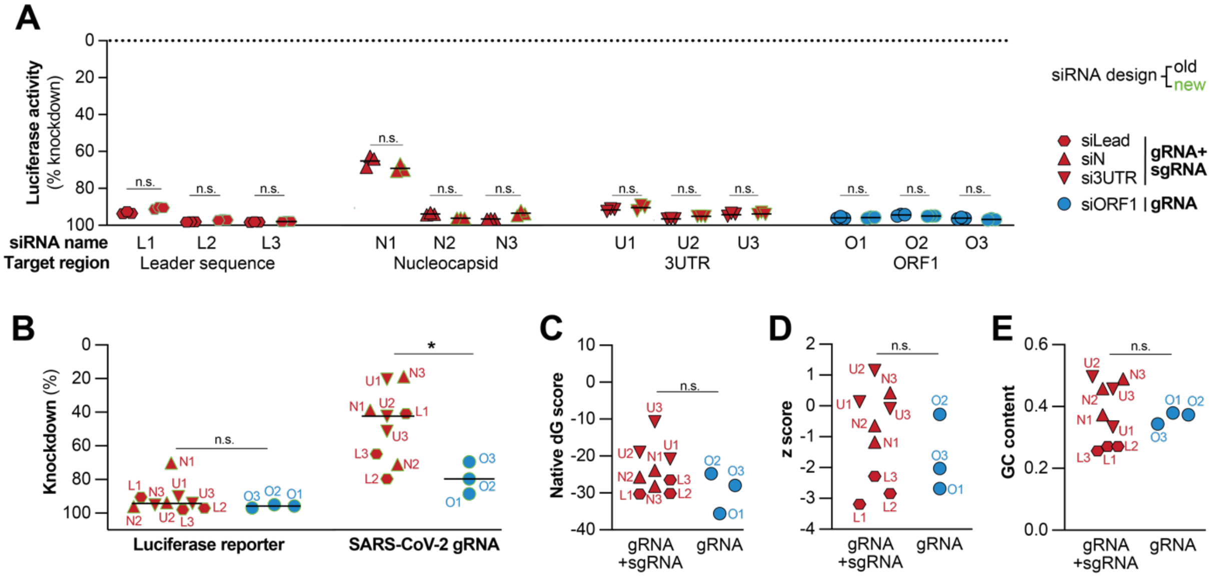

Supplementary Figure S6. Effect of siRNA design and RNA secondary structure on antiviral activity of siRNAs. (**A**) Activity of original siRNAs which slightly varied in length and the containment of wobbles (old design, see Table 1) were compared to siRNAs which all had identical length and no wobbles (new design, see Supplementary Table S3) using luciferase reporters. siRNAs (10 nM) and plasmids encoding for luciferase reporters were co-transfected into HEK293T cells and luciferase activity measured after 48 h. (**B**) Antiviral activity of siRNAs was evaluated by transfecting siRNAs (1 nM) into VeroE6 cells, which were infected with rSARS-CoV-2-GFP and reduction of GFP^{+} cells assessed 24 h p.i. (**C-E**) Predicted secondary structures of siRNA target regions was analyzed by using scan-fold results (provided by Andrews et al. (49)). (**C**) Native dG score (or 'minimum free energy' [MFE]) predicts the free energy value of the most stable structure the sequence could adopt. A more negative value represents a more stable structure. (**D**) Thermodynamic Z-score compares the minimum free energy of the native sequence to a scrambled version (negative z-score indicating a stable structure), and (**E**) the GC content positively correlates with stable secondary structures (for details see material & methods).

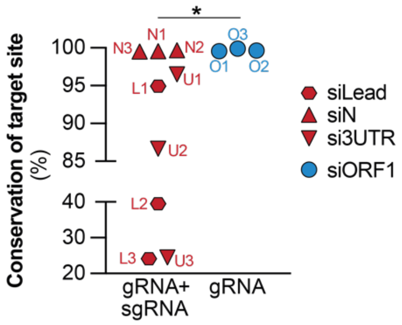

Supplementary Figure S7. Comparison of the target site conservation between siRNAs targeting ORF1 or the common region of transcripts. Full-length, high-quality SARS-CoV-2 sequencing results were retrieved from the GISAID EpiCoV™ Database (www.gisaid.org). The latest 100,000 submissions before October 26th 2021 were downloaded (without restricting to a specific variant) and analyzed for the presence of the siRNA target sites. Only perfect matches were counted. Statistical difference was calculated using Mann-Whitney U Test. *, $p<0.05$

**Name**	**Sense strand (5'-3')**	**Antisense strand (5'-3')**	**Length** (nt)	
			Sense	Antisense
**O1***	[C]*-[C]*-[A]-[A]-[A]-[U]-FluoroG-[U]-FluoroG-FluoroC-FluoroC-[U]-[U]-[U]-[C]-[A]-[A]-[C]-[U]	[A]*-FluoroG*-[U]-[U]-[G]-FluoroA-[A]-FluoroA-FluoroG-[G]-[C]-[A]-[C]-FluoroA-[U]-FluoroU-[U]-[G]-[G]*-[U]*-[U]	19	21
**O2***	[G]*-[U]*-[U]-[A]-[C]-[A]-FluoroU-[G]-FluoroC-FluoroA-FluoroC-[C]-[A]-[U]-[A]-[U]-[G]-[G]-[A]	[U]*-FluoroC*-[C]-[A]-[U]-FluoroA-[U]-FluoroG-FluoroG-[U]-[G]-[C]-[A]-FluoroU-[G]-FluoroU-[A]-[A]-[C]*-[A]*-[A]	19	21
**O3***	[G]*-[G]*-[U]-[A]-[C]-[U]-FluoroU-[G]-FluoroG-FluoroU-FluoroA-[G]-[U]-[U]-[U]-[A]-[G]-[C]-[U]	[A]*-FluoroG*-[C]-[U]-[A]-FluoroA-[A]-FluoroC-FluoroU-[A]-[C]-[C]-[A]-FluoroA-[G]-FluoroU-[A]-[C]-[C]*-[A]*-[U]	19	21
**siGFP***	[G]*-[C]*-[A]-[G]-[C]-[A]-FluoroC-[G]-FluoroA-FluoroC-FluoroU-[U]-[C]-[U]-[U]-[C]-[A]-[A]-[G]	[C]*-FluoroU*-[U]-[G]-[A]-FluoroA-[G]-FluoroA-FluoroA-[G]-[U]-[C]-[G]-FluoroU-[G]-FluoroC-[U]-[G]-[C]*-[U]*-[U]	19	21
**siLuc***	[C]*-[G]*-[U]-[A]-[C]-[G]-FluoroC-[G]-FluoroG-FluoroA-FluoroA-[U]-[A]-[C]-[U]-[U]-[C]-[G]-[A]	[U]*-FluoroC*-[G]-[A]-[A]-FluoroG-[U]-FluoroA-FluoroU-[U]-[C]-[C]-[G]-FluoroC-[G]-FluoroU-[A]-[C]-[G]*-[U]*-[G]	19	21

Supplementary Table S4. Sequences and design pattern of chemically modified siRNAs used in the study. siRNA dublexes were designed with an asymmetric design. nt, nucleotides; GFP, Green Fluorescent Protein; Luc, Firefly Luciferase; *, Phosphorothioate linkage; [], 2'-O-Methyl modification; Fluoro, 2' Fluoro modification; A, Adenine; C, Cytosine; G, Guanine; U, Uracil.
